# Improving existing analysis pipeline to identify and analyze cancer driver genes using multi-omics data

**DOI:** 10.1038/s41598-020-77318-1

**Published:** 2020-11-25

**Authors:** Quang-Huy Nguyen, Duc-Hau Le

**Affiliations:** 1 Department of Computational Biomedicine, Vingroup Big Data Institute, Hanoi, Vietnam; 2 Faculty of Pharmacy, Dainam University, Hanoi, Vietnam; 3 College of Engineering and Computer Science, VinUniversity, Hanoi, Vietnam

**Keywords:** Computational biology and bioinformatics, Data integration

## Abstract

The cumulative of genes carrying mutations is vital for the establishment and development of cancer. However, this driver gene exploring research line has selected and used types of tools and models of analysis unsystematically and discretely. Also, the previous studies may have neglected low-frequency drivers and seldom predicted subgroup specificities of identified driver genes. In this study, we presented an improved driver gene identification and analysis pipeline that comprises the four most widely focused analyses for driver genes: enrichment analysis, clinical feature association with expression profiles of identified driver genes as well as with their functional modules, and patient stratification by existing advanced computational tools integrating multi-omics data. The improved pipeline's general usability was demonstrated straightforwardly for breast cancer, validated by some independent databases. Accordingly, 31 validated driver genes, including four novel ones, were discovered. Subsequently, we detected cancer-related significantly enriched gene ontology terms and pathways, probable drug targets, two co-expressed modules associated significantly with several clinical features, such as number of positive lymph nodes, Nottingham prognostic index, and tumor stage, and two biologically distinct groups of BRCA patients. Data and source code of the case study can be downloaded at https://github.com/hauldhut/drivergene.

## Introduction

Cancer is one of the most dangerous diseases that poses as a threat to public health, second only to behind cardiovascular disease^1^. In recent years, we have gradually realized that genes carrying mutations are critical for the establishment and development of cancer^2–6^. It has rung the warning bell for cancer researchers to rapidly identify and characterize driver genes in each cancer type that will make enormous contributions to precision medicine in cancer treatment in the future.

Over the past five years, however, researchers tend to disagree on a unifying pipeline for analysis^7–15^. This was likely because they were used to solve specific problems regarding the cancer of interest; thus, there was no common direction that has been proposed to apply for most types of cancers. For example, iCAGES^16^ was used to identify driver genes and personalized treatments for clear cell renal cell carcinoma^10^, and then the identified driver genes were enriched with gene ontology terms and biological pathways using Gene Ontology^17^ and STRING^18^, respectively. Besides, a co-expression network was constructed with the WGCNA package^19^ to analyze the association between co-expressed modules and clinical features. For prostate cancer^11^, a set of driver identification tools such as OncodriveCLUST^20^, OncodriveFM^21^, iCAGES^16^ and DrGaP^22^ were used. Then, Gene Ontology^17^ and STRING^18^ were employed to annotate the identified driver genes. In addition, WGCNA^19^ was used to identify the co-expressed module-clinical feature association. For kidney cancer^7^, driver genes and their cancer pathways were predicted with OncodriveFM^21^ and OncodriveCLUST^20^, and then assessed for the association between high-scoring noncoding variants and their regulatory features using three computational tools such as CADD^23^, FunSeq2^24^ and GWAWA^25^. For hepatocellular carcinoma^8^, the identification of driver genes and their pathways was performed with OncodriveFM^21^, Dendrix^26^, and the enrichment analysis was additionally performed with Gene Ontology^17^ and STRING^18^ data. Finally, Kaplan–Meier survival analyses were done using OncoLnc^27^ to observe an association between survival rates and each driver gene.

In addition, previous studies have paid close attention to recurrently mutated genes and coding driver genes in cancer patients by using high-frequency-specific tools such as MutSigCV^28^ and MuSiC^29^. However, plenty of cancer drivers was mutated at less than 1% of cancer patients^14,30^. Therefore, previous results may have neglected rare cancer drivers. Since cancer is heterogeneous, stratifying cancer patients with the identified driver genes is a core element in the development of precision medicine for tackling this heterogeneity. However, the previous studies have been almost rarely touched on the taxonomy of cancer patients using somatically mutated genes. This raises a tough challenge to suggest specifically clinical guidance for each cancer patient, except for some recent case studies like prostate cancer^11^, breast cancer (BRCA)^13,14^. Even ref.^11^ and ref.^14^ used a hierarchical clustering method at a basic level that can totally be improved. In addition, ref.^11^ also did not clarify how they identified the number of classes of patients and whether the number is optimal.

Our study was collectively developed based on previous studies to overcome the above challenges, using a case study of breast cancer from the popular database METABRIC^13^. The goal of this work is to help improve the available analysis pipeline, in a more systematic and efficient way, in the identification and analysis of driver genes in future studies. Moreover, in recent years, developments on multi-omics data integration related to BRCA have been useful and efficient in various aspects^31–35^. This is the basis for us to propose the pipeline relying on integrative multi-omics implementation. For this purpose, in the real data analysis, a total of 35 driver genes was predicted using somatic mutation data, and then 31 driver genes closely related to BRCA were validated and used for subsequent analyses. They were first significantly enriched with gene ontology terms and pathways. The associations between the identified driver genes and their two co-expressed modules with several clinical features such as survival rate, number of positive lymph nodes, Nottingham prognostic index, and cancer stage were also analyzed using gene expression data. Finally, BRCA patients were stratified into two distinct subgroups using copy number data of the identified driver genes with significant differences concerning the clinical features.

## Material and methods

### Overview of an improved pipeline

**Figure 1:**
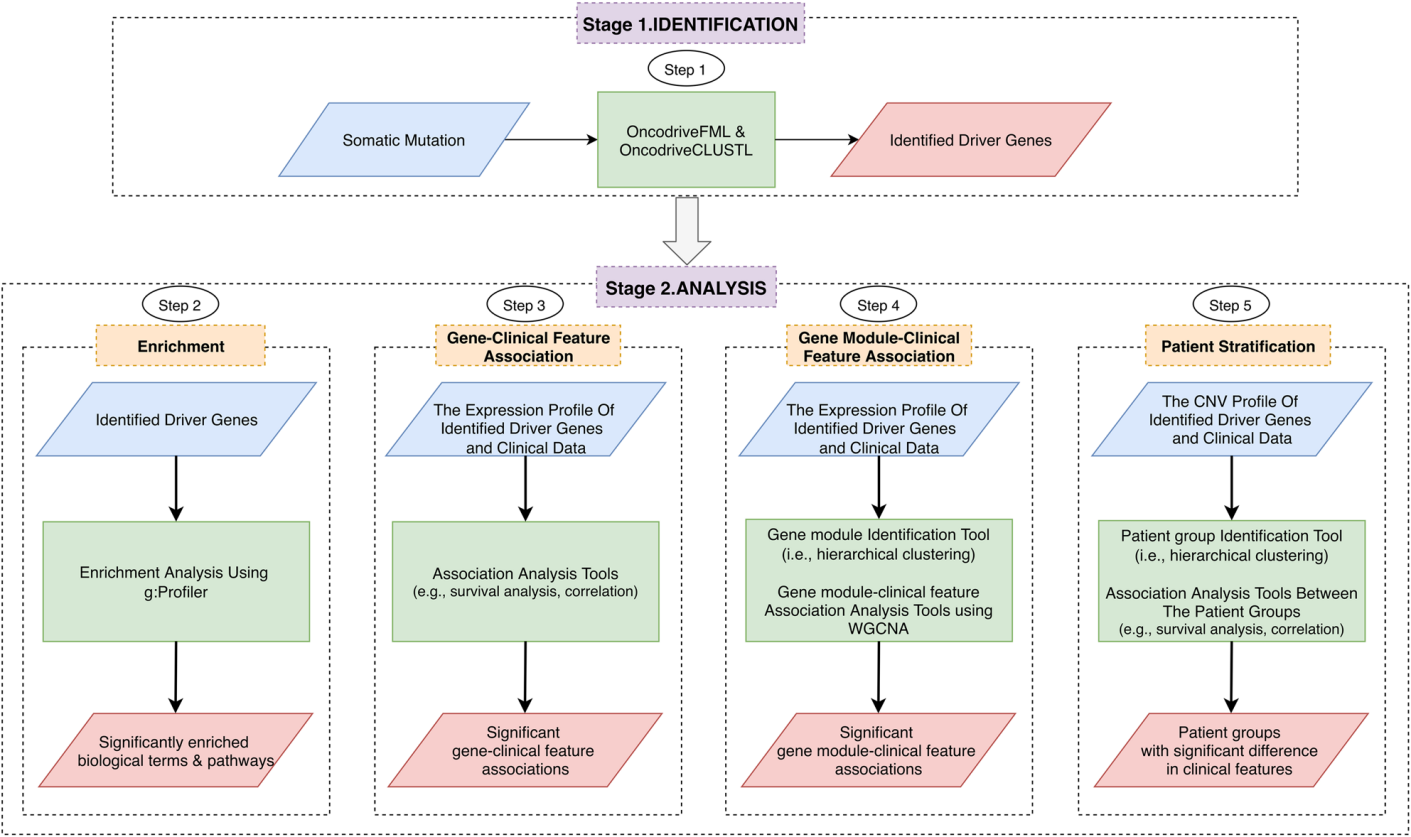
Improved analysis pipeline for identification and analysis of driver genes. The scheme comprises two stages: identification and analysis, in which the former uses the OncodriveFML and OncodriveCLUSTL to identify driver genes with somatic mutation data as input, and the latter performs the four most widely focused analyses to deal with those driver genes. Abbreviation: CNV, Copy number variations.

Figure 1 illustrates the improved analysis pipeline to identify and analyze cancer driver genes. The scheme is conceptually straightforward with two stages: identification and analysis. For the identification stage, somatic mutation data is inputted to identify driver genes using the OncodriveCLUSTL^36^ and OncodriveFML^37^ tools (Step 1). For the analysis stage, we provide the four most widely focused analyses in the cancer driver exploring studies^7–15^. Firstly, the identified driver genes will be annotated by the tool g:Profiler^38^ (Step 2). Secondly, they are further investigated for the association between expression levels of genes and clinical features of interest by statistical tools (Step 3). Thirdly, the association of their functional modules (e.g., co-expressed modules, which are identified with a hierarchical agglomerative clustering method^39^) with the clinical features is also performed by the tool WGCNA^19^ (Step 4). Fourthly, patients can be stratified into subgroups on the basis of the copy number profiles of the identified driver genes using a hierarchical agglomerative clustering method^39^ (Step 5).

### List of improvements proposed in the work

#### Selection of driver gene prediction tools

This work recommends the use of OncodriveFML and OncodriveCLUSTL packages for predicting driver genes. In fact, according to the previous studies, OncodriveFM and OncodriveCLUST tools were employed many times^7,8,11^. However, a potential weakness of the OncodriveFM compared to the OncodriveFML is only the detection of coding driver genes, whereas the OncodriveCLUST also has several weaknesses solved by the OncodriveCLUSTL, like disregarding drivers whose mutations are distributed across the sequence, or requiring a large quantity of observed mutations to ensure a good outcome. From that, the OncodriveFML and OncodriveCLUSTL packages are proven as state-of-the-art methods, even confirmedly outperforming the above two^36,37^. Moreover, they can not only help researchers find low-frequency and non-coding mutated genes but also were designed as friendly web-based applications.

#### Selection of enrichment tools

To understand in-depth the underlying biological phenomenon, the enrichment analysis is performed to discover involved biological processes and pathways of identified driver genes. This study recommends the use of g:Profiler package^38^ for enrichment analysis since it is one of the rich-annotated, friendly web-based, and up-to-date gene enrichment tools. Also, it was recommended to use for enrichment analysis step in a protocol proposed in^40^. Otherwise, as most previous cancer-driver-exploring studies^7–11^, Gene Ontology and STRING are both popular annotation resources and tools; thus, users may consider selecting them as alternative tools. Besides, users can also select other alternative options, such as GSEA^41^, DAVID^42,43^, IPA^44^, etc. Although they are advanced, but have their own weaknesses. For instance, the biggest weakness of the GSEA and IPA tools is that they are bulky software which require the user to run as a desktop application. Moreover, the IPA software is a commercial one. In contrast, DAVID is a web-based, but rarely updated tool.

#### Association analysis of individual genes with clinical features

There are several available correction methods for multiple testing (e.g., Hochberg’s method^45^, Bonferroni correction^46^, Holm’s method^47^, etc.) and correlation methods (i.e., Pearson, Spearman’s rank, Kendall, etc.); consequently, the previous studies selected and computed adjusted *P*-values (i.e., Q-values) and correlation coefficients unclearly, resulting in making the results ambiguous and running into difficulty in reproducing them. For example, ref.^11^ did not indicate specifically which *P*-value adjustment method was chosen. To produce consistent results and be convenient for users, we now built the R package ‘computeC’ (https://github.com/huynguyen250896/computeC), which computes correlation coefficients between each detected driver versus each clinical feature of interest. Then, the obtained *P*-values are automatically adjusted by the Benjamini–Hochberg procedure^48^. The problem with Bonferroni, Hochberg and Holm is that they are correction methods for a small number of tests (*n*). If the sample size *n* is large, they will reject too many null hypotheses. From that, Benjamini and Hochberg's false discovery rate (FDR) may be a better choice. Likewise, many previous works^49–53^ related the expression levels of each identified driver gene to prognostic value (e.g., the overall survival of patients), and the genes when *P*-value ≤ 0.05 (Log-rank test) were considered to define significant association. Again, FDR control is crucial, so we developed the tool ‘geneSA’ (https://github.com/huynguyen250896/geneSA) to automatically do the above task and only preserve the genes if Q-value ≤ 0.05 (Benjamini–Hochberg FDR).

#### Selection of tools for unsupervised identification of co-expressed gene modules and patient groups

Most co-expression network construction tools are based on unsupervised methods. Besides, as mentioned above, previous studies have not paid enough focus on driver gene-based patient stratification, which may make critical contributions to the design of therapeutic strategies^13^. Indeed, there is lots of evidence reporting that individual driver genes are mutated predominantly in the samples within one single subtype than in the others, suggesting that those driver genes are recognized as subtype-specific driver genes^54^. Those driver genes then serve as the important clues to monitor the difference among the explored subtypes^54,55^ as well as help to develop personalized treatments^56^. To this end, this study recommends the use of a hierarchical agglomerative clustering method for co-expressed module identification (Fig. 1, Stage 2—Step 4) and patient stratification (Fig. 1, Stage 2—Step 5), which is a common selection from previous studies^10,11,14,49,51,52^. Furthermore, these previous works performed the hierarchical clustering method using complete linkage and Euclidean distance for the clustering task in co-expression network construction and patient stratification. The work recommends adding a sub-step in this process: selecting the best agglomeration method, which designates how a hierarchical clustering method clusters objects (see the ‘Supplementary File 1′ section for the implementation). When it comes to the agglomerative coefficient, it measures the number of clustering structures found (values closer to 1 suggest strong clustering structure) and specifies the agglomeration method to be used (i.e., one of ‘complete’, ‘average’, ‘single’, or ‘ward’). More specifically, the agglomeration methods Complete/Average/Single-linkage first compute pairwise dissimilarities of the objects in group 1 and group 2. Then, those methods treat the maximum/mean/minimum value of the calculated dissimilarities as the distance between the involved groups, respectively. In contrast, Ward’s minimum variance method first minimizes the total within-cluster error sum of squares, and then, at each stage, iteratively identifies pairs of groups with minimum between-group distance and do the fusion of those two.

#### Selection of cluster validation

This process aims to assess the quality of the clustering results^57^ and determine the success or failure of the clustering works^58^. This work recommends the user to select the Dunn’s index^46,59^. This is an efficient cluster validation method, and Curtis et al.^60^ used it to validate the detected subgroups of patients with BRCA from METABRIC. Furthermore, to increase the reliability and robustness of results, the user should combine two or three methods at once. This study additionally recommends the user to select the average Silhouette index^61^ for two main reasons. First, it is among the best clustering indices^62^. Second, it was suggested to use in the clustering task of biomedical data^63^. For instance, it was selected as the index for comparing the quality of clustering results between clustering methods for breast cancer^64^.

### Case study: breast cancer

#### Datasets and data preprocessing

The BRCA data were downloaded from the cBioPortal for Cancer Genomics (https://www.cbioportal.org)^65^. It contained the METABRIC BRCA cohort assembled from 2509 primary breast cancer patients with 548 matched normals in the United Kingdom and Canada^13^. The gene expression microarray data were generated using the Illumina Human v3 microarray for 1904 samples, while the CNVs data were measured on the Affymetrix SNP 6.0 platform for 2173 samples. In addition, 17,272 somatic mutations of 173 genes for 2369 samples were detected on the Illumina HiSeq 2000 platform. R scripts responsible for the work's implementation are provided in the Github repositories (https://github.com/hauldhut/drivergene) (See more detail in Supplementary File 1).

All omics data we pre-processed in the same way as in the reference paper^13^. Specifically, we only matched the sample labels shared between the gene expression data and clinical data, and the CNVs data and clinical data, and obtained 1904 and 2173 matched patients, respectively.

#### Identification of driver genes using OncodriveFML and OncodriveCLUSTL

In the first stage, we used two driver identification tools: OncodriveFML and OncodriveCLUSTL (Fig. 1, stage 1; Step 1) to detect potential cancer-related genes of BRCA. OncodriveCLUSTL 1.1^36^ is a sequence-based clustering algorithm to identify significant clustering signals of mutations across genomic regions; meanwhile, OncodriveFML 1.0^37^ is a method designed to analyze somatic mutations across cancer samples to positively select driver genes (i.e., Detection of positive selection in cancer genomes is to identifying genes essential for cancer growth^66^). Both of them are able to detect cancer driver genes in both coding regions and non-coding regions as well as non-human data. The parameters were set to default values with the exception of the sequencing parameter (targeted sequencing) and the scoring system CADD v1.3^23^ (the latest version at the time of this writing) in the OncodriveFML tool and the selection of ‘concatenate’ option in the OncodriveCLUSTL. A gene is considered a potential driver gene identified by the OncodriveFML and OncodriveCLUSTL when Q-value < 0.25 (Figure S2, Supplementary File 1) and Q-value < 0.01 (Figure S3 Supplementary File 1) (Benjamini–Hochberg FDR), respectively. Then, according to the Cancer Gene Census database (CGC; https://cancer.sanger.ac.uk/census)^67^, Pereira et al. reference paper^13^ and Nik Zainal et al*.* paper^14^, we verified those identified driver genes. The CGC database provides a list of genes, which have been common in cancer development. Finally, driver genes were said to be *bona fide* ones if they met the validation process and were served for downstream analysis.

#### Enrichment analysis using g:Profiler

To deepen our understanding of the potential biological functions of our BRCA-associated genes, the enrichment analysis was performed using g:Profiler to discover involved biological processes and pathways. More specifically, among provided annotation resources, biological process (BP) terms, under the sub-tab ‘Gene Ontology’ (GO), and KEGG pathways, under the sub-tab ‘biological pathways’, were chosen to characterize the identified driver genes functionally. GO terms and pathways were considered to be significantly enriched if a cut-off of Q-value ≤ 0.05 (g:SCS multiple testing correction method).

#### Individual gene-clinical feature association analysis

In this section, we analyzed associations between clinical features of interest and the identified driver genes. More specifically, gene expression data were used to examine the associations between individual drivers and several familiar clinical features, including survival rates^11,13,15,49^, numbers of positive lymph nodes^11,13^, Nottingham prognostic index, and pathologic stages^11,15^ of 1,904 patients.

For survival rate, we performed a survival analysis for the expression profiles of each driver gene, like in refs.^49–53^. In brief, given a driver gene, the median expression of that gene was calculated across the patients, then the patients were classified into two groups based on the expression of the gene. The first group ‘up-regulation’ includes patients having the expression of the genes was greater than the median; meanwhile, the second group ‘down-regulation’ includes patients having the expression of the genes was less than the median. Then, a log-rank test in univariate Cox regression analysis with a proportional hazards model^68^ (i.e., implemented by the ‘geneSA’ function) was used to compare the survival rates between the two groups. Next, hazard ratios (HR) with their 95% confidence intervals (CI), *P*-values, and Q-values were reported. Driver genes were considered to be significantly associated with survival rate if Q-value ≤ 0.05. Finally, we validated those prognostic driver genes by using KMplot website (https://kmplot.com/analysis/index.php?p=service&start=1)^69^ with the ‘Survival’ option of ‘OS (n = 1402)’ and the ‘Split patients by’ option of ‘median’, the remaining options were left at default.

Additionally, for the three clinical features (i.e., the number of positive lymph nodes, the Nottingham prognostic index and the pathologic stages), we correlated them with the expression of each driver gene using Spearman's rank correlation method (i.e., implemented by the ‘computeC’ function in R). Driver genes were considered to be significantly associated with positive lymph nodes, Nottingham prognostic index, or pathologic stages if Q-value ≤ 0.05.

#### Co-expressed module-clinical feature association analysis using WGCNA

We first found the optimal soft threshold β to make the co-expression matrix of the identified driver genes fit a scale-free topology model, then the Topological Overlap Matrix (TOM)-based dissimilarity matrix of the identified driver genes was computed using Pearson’s correlation (see Supplementary File 1: User manual). Next, the co-expressed modules were detected by two steps: (1) an agglomerative clustering algorithm, i.e., Ward’s method^70^, implemented by R function ‘hclust’ in the package ‘flashClust’^71^ was used to hierarchically cluster the TOM-based dissimilarity matrix into a gene dendrogram; (2) Then, those genes were distributed to each resulting module with the minimum number of genes was set as ten by the function ‘cutreeDynamic’ implemented in the package ‘dynamicTreeCut’^72^. Notably, to make the gene network consistent, according to prior studies^10,11,53^, we suggest that users should choose the ten number of genes existing in each module minimally. Genes with high intra-modular connectivity were considered as hub genes. The association between resulting co-expressed modules and the clinical features was then analyzed using the correlation between the modular eigengene and the clinical features.

#### Patient stratification

Similar to step 4 (Fig. 1, stage 2), hierarchical agglomerative clustering of all the patients using Ward’s method and Euclidean distance creates a patient dendrogram. Then, the function ‘clValid’^73^ reports how many patient groups were optimal by connectivity, Dunn’s index, and Average Silhouette algorithm, and the BRCA patients were distributed to each group using the ‘cutree’ with ‘agnes’ functions. To observe the differences, we further performed the analyses between groups in terms of the clinical features. For survival rate, the log-rank test in univariate Cox regression analysis with a proportional hazards model was employed to compare the survival rates of the patients between the involved groups, and Kaplan–Meier curves were then plotted by the R package ‘survminer’^74^. In addition, the number of positive lymph nodes, the Nottingham prognostic index, and the tumor stages were also compared between the discovered groups. Subsequently, we tested the significance between the given results in terms of these clinical features using the function ‘compareGroups’^75^. *P*-value ≤ 0.05 was predefined as statistically significant.

## Results

### Identification of driver genes using OncodriveFML and OncodriveCLUSTL

**Figure 2:**
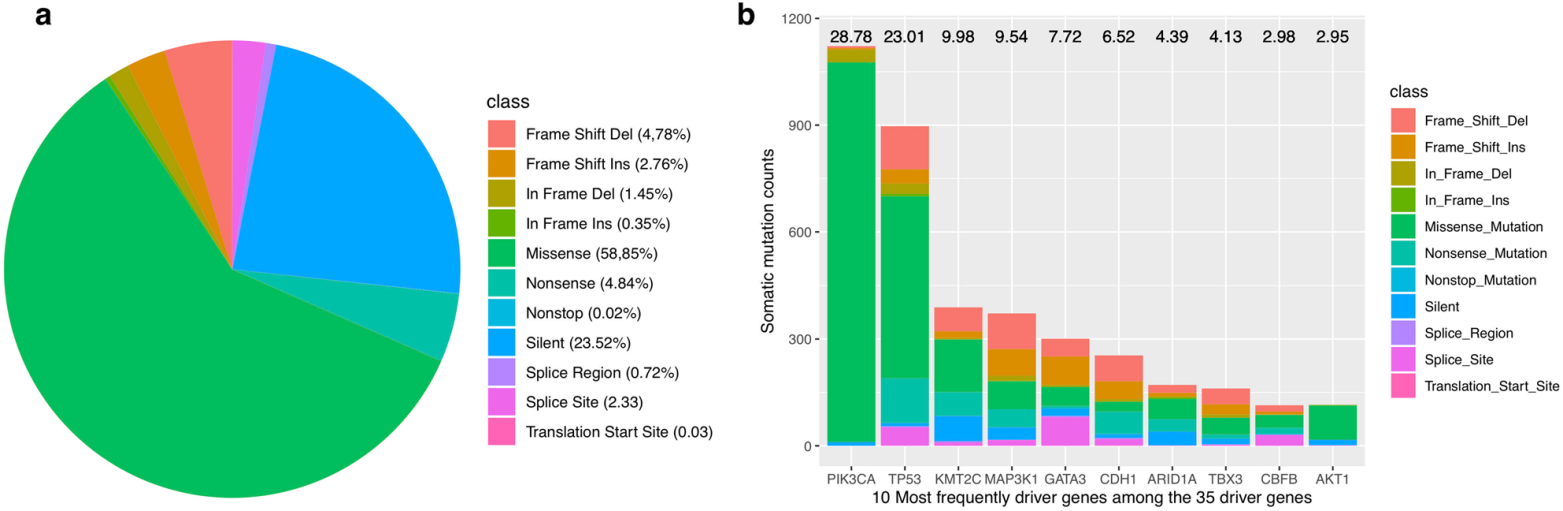
Characterization of somatic mutations and driver genes in BRCA. (**a**) Summarization of mutation classes in BRCA. (**b**) Number and rate of the ten most frequently mutated driver genes.

Pathological clinical features (e.g., survival time, tumor stage, number of lymph nodes, and Nottingham prognostic index) were collected (Table 1). All of the 17,272 somatic mutations were used as input to both of the tools. These mutations include 10,165 missense (non-synonymous mutations), 4,063 silent, 402 splice-site, 836 nonsense, 124 splice-region, 5 translation start site, 4 nonstop mutations, and 1,613 insertions or deletions (indels) (Fig. 2a). Of the 1,613 indels, 1,302 is frameshift, 311 is in-frame. A total of 35 unique driver genes were detected by the two tools, in which 30 and 10 driver genes were predicted by OncodriveFML and OncodriveCLUSTL, respectively (Supplementary File 2, Table S1). Both of the tools detected five of them simultaneously, including *AKT1, CDH1, ERBB2, ERBB3,* and *TP53*. Among the 35 driver genes, *PIK3CA, TP53, KMT2C, MAP3K1, GATA3, CDH1, ARID1A, TBX3, CBFB* and *AKT1* were the 10 most frequently mutated genes in BRCA, with mutation rates of 28.78%, 23.01%, 9.96%, 9.54%, 7.72%, 6.52%, 4.39%, 4.13%, 2.98% and 2.95%, respectively (Fig. 2b). Interestingly, some of the involved genes were lowly mutated ones in BRCA, including *CDKN2A*, 0.6% and *KRAS*, 0.6% (we checked mutation frequencies through cBioPortal website^65^).

**Table 1:** Description of the clinical features of the patients included in the study.

Clinical features	Overall study cohort (n = 2509)		Data type
	n	%	
Tumor stage			Nominal variable
0	24	1.0	
1	630	25.1	
2	979	39.0	
3	144	5.8	
4	11	0.4	
Unknown	721	28.7	
Number of positive lymph nodes			Continuous variable
0—≤ 3	695	27.7	
> 3—≤ 9	233	9.3	
> 10	119	4.7	
Unknown	1462	58.3	
Nottingham prognostic index			
1—≤ 2.4	219	8.7	
> 1—≤ 3.4	555	22.1	
> 3.4—≤ 5.4	1256	50.1	
> 5.4	252	10.0	
Unknown	227	9.1	
Follow-up months, median*	116.47 ± 76.11		Survival
Survival status			
Alive (= 0)	837	33.4	
Death (= 1)	1144	45.6	
Unknown	528	21.0	

*Missing information is excluded.

By comparing the predicted driver genes to TSGene (i.e., a database of tumor suppressor genes; Supplementary File 2, Table S2)^76^ and ONGene (i.e., a database of oncogenes; Supplementary File 2, Table S3)^77^, this study detected 13 tumor suppressor genes including *MAP2K4, ARID1A, TP53, PTEN, CDH1, NF1, RB1, CDKN2A, FOXO3, SMAD4, BRCA2, BAP1, and MEN1* as well as 11 known oncogenes including *PIK3CA, TBX3, CBFB, AKT1, RUNX1 CDH1, PIK3R1, CDKN1B, ERBB2, ERBB3,* and *KRAS*. According to the CGC database and two in vitro experiments^13,14^, we realized that 31 out of 35 driver genes were genuine (Supplementary File 2, Table S1). On top of that, to the best of our knowledge, several genes, including *AHNAK, DNAH2, PDE4DIP,* and *SYNE1* were detected as driver genes in BRCA for the first time (Supplementary File 2, Table S1).

### Enrichment analysis using g:Profiler

As a result, 483 biological processes (Supplementary File 2, Table S4), and 71 pathways (Supplementary File 2, Table S5) were significantly overrepresented for the gene set. Tables 2 and 3 show ten most enriched biological processes and ten most enriched KEGG pathways, respectively. The majority of biological process terms and pathways were widely known as cancer-related, such as “negative regulation of developmental process”^34,78^, “positive regulation of gene expression”, “regulation of gene expression”^34^,… for biological processes, whereas for KEGG pathways, cancer-specific ones were ‘Chronic myeloid leukemia’, “Endometrial cancer”, “Non-small cell lung cancer”, … These results further confirmed that cancer driving-genes detected by the two tools OncodriveFML and OncodriveCLUSTL have key functions in cancer in general and breast cancer in particular.

**Table 2:** Ten most enriched gene ontology terms. Q-value is computed by using g:SCS multiple testing correction method. Abbreviation: Num, Number of genes involving in the term.

GO ID	Term name	Num	Q-value
GO:0010604	Positive regulation of macromolecule metabolic process	29	3.44 × 10^–15^
GO:0009893	Positive regulation of metabolic process	29	3.14 × 10^–14^
GO:0051173	Positive regulation of nitrogen compound metabolic process	27	2.38 × 10^–13^
GO:0051093	Negative regulation of developmental process	19	7.33 × 10^–13^
GO:0010628	Positive regulation of gene expression	24	8.55 × 10^–13^
GO:0031325	Positive regulation of cellular metabolic process	27	9.02 × 10^–13^
GO:0045596	Negative regulation of cell differentiation	17	1.22 × 10^–12^
GO:0048513	Animal organ development	27	5.77 × 10^–12^
GO:0048518	Positive regulation of biological process	31	1.97 × 10^–11^
GO:0010468	Regulation of gene expression	29	4.16 × 10^–11^

**Table 3:** Ten most enriched KEGG pathways. Q-value is computed by using g:SCS multiple testing correction method. Abbreviation: Num, Number of genes involving in the pathway.

Pathway ID	Pathway name	Num	Q-value
KEGG:05213	Chronic myeloid leukemia	10	5.02 × 10^–12^
KEGG:01522	Endometrial cancer	9	2.24 × 10^–11^
KEGG:05223	Endocrine resistance	10	5.67 × 10^–11^
KEGG:05218	Non-small cell lung cancer	9	1.01 × 10^–10^
KEGG:05166	Melanoma	9	1.73 × 10^–10^
KEGG:01521	Human T-cell leukemia virus 1 infection	12	3.62 × 10^–10^
KEGG:05215	EGFR tyrosine kinase inhibitor resistance	9	4.12 × 10^–10^
KEGG:05226	Prostate cancer	9	2.74 × 10^–09^
KEGG:04218	Gastric cancer	10	4.55 × 10^–09^
KEGG:05213	Cellular senescence	10	7.69 × 10^–09^

### Individual gene-clinical feature association analysis

As a result, we found nine genes, including *AKT1, KMT2C, KRAS, PIK3R1, PTEN, SMAD4, MAP3K1, MAP2K4* and *TBX3*, significantly correlated with the survival rate (Supplementary File 2, Table S6). Through the validation process using the KMplot database (Supplementary File 1, Figure S4), we realized that five out of nine driver genes were prognostic ones that we may be interested in best (Table 4). Among them, *KRAS* with up-regulation levels, and four other genes, including *MAP2K4, MAP3K1, PIK3R1,* and *TBX3* with down-regulation levels significantly associated with shortened lifespan (down-regulation is the reference) (Table 4). This suggested that those driver genes were related to the pathophysiology of breast cancer in varying degrees. They could also help observe the rigor of breast cancer or anticipate the survival rate of patients.

**Table 4:** Validated association between the expression of driver genes and the overall survival of BRCA patients.

Gene	HR (95% CI)	*P*-value	Q-value
KRAS	1.20 (1.07–1.35)	2.30 × 10^–03^	1.19 × 10^–02^
MAP2K4	0.76 (0.67–0.85)	4.57 × 10^–06^	1.42 × 10^–05^
MAP3K1	0.82 (0.73–0.93)	1.23 × 10^–03^	7.61 × 10^–03^
PIK3R1	0.84 (0.75–0.95)	4.37 × 10^–03^	1.93 × 10^–02^
TBX3	0.84 (0.75–0.95)	4.91 × 10^–03^	1.90 × 10^–02^

One gene, including *KRAS* with above-median expression levels and four genes, including *MAP2K4, MAP3K1, PIK3R1,* and *TBX3* with below-median expression levels significantly associated with a shortened lifespan. HR is a measure that helps determine whether either of two expression levels of each driver gene will result in an increased (i.e., HR > 1) or decreased (i.e., HR < 1) probability of experiencing the defined event (i.e., death), at any time (below-median expression level is the reference). *P*-value is computed by the Cox proportional hazard method to test the statistical difference of the given results. Q-value is computed following the Benjamini–Hochberg procedure. HR: hazard ratio. 95% CI: 95% confidence interval.

For the three clinical features (i.e., the number of positive lymph nodes, the Nottingham prognostic index, and the pathologic stages), there are a large number of driver genes negatively correlated with the number of positive lymph nodes (12 genes; A, Supplementary File 2, Table S7), Nottingham prognostic index (16 genes; Supplementary File 2, Table S8), and the pathologic stages (10 genes, Supplementary File 2, Table S9). Similarly, several genes are positively correlated with the number of positive lymph nodes (four genes; Supplementary File 2, Table S10), the Nottingham prognostic index (seven genes; Supplementary File 2, Table S11), and the pathologic stages (three genes; Supplementary File 2, Table S12). A total of 10 genes, including *ARID1A, RUNX1, GATA3, TBX3, NF1, MAP2K4, PTEN, SMAD4, MAP3K1,* and *SF3B1* showed significant associations with all of the three clinical features (Table 5).

**Table 5:** Association between the expression of driver genes and the other clinical features.

Gene	Number of lymph nodes			Nottingham prognostic index			Cancer Stage		
	CC	*P*-value	Q-value	CC	*P*-value	Q-value	CC	*P*-value	Q-value
ARID1A	− 0.06	0.01	0.02	− 0.13	1.31 × 10^–8^	3.69 × 10^–8^	− 0.10	1.13 × 10^–4^	5.82 × 10^–4^
RUNX1	− 0.14	1.65 × 10^–9^	5.12 × 10^–8^	− 0.25	6.20 × 10^–29^	9.61 × 10^–28^	− 0.11	2.97 × 10^–5^	4.61 × 10^–4^
GATA3	− 0.01	1.27 × 10^–5^	1.31 × 10^–4^	− 0.28	1.28 × 10^–35^	3.98 × 10^–34^	− 0.12	3.83 × 10^–5^	3.95 × 10^–4^
TBX3	− 0.10	9.10 × 10^–6^	1.41 × 10^–4^	− 0.18	1.44 × 10^–15^	7.41 × 10^–15^	− 0.12	6.12 × 10^–5^	4.74 × 10^–4^
NF1	− 0.09	5.77 × 10^–5^	4.47 × 10^–4^	− 0.08	2.23 × 10^–4^	3.64 × 10^–4^	− 0.07	6.36 × 10^–3^	0.02
MAP2K4	− 0.08	4.61 × 10^–4^	1.79 × 10^–3^	− 0.22	2.04 × 10^–21^	1.58 × 10^–20^	− 0.07	6.99 × 10^–3^	0.02
PTEN	− 0.08	6.02 × 10^–4^	2.07 × 10^–3^	− 0.23	1.22 × 10^–23^	1.26 × 10^–22^	− 0.11	2.93 × 10^–5^	9.08 × 10^–4^
SMAD4	− 0.06	0.01	0.03	− 0.10	1.79 × 10^–5^	3.47 × 10^–5^	− 0.08	1.48 × 10^–3^	5.11 × 10^–3^
MAP3K1	− 0.06	0.01	0.03	− 0.16	6.35 × 10^–13^	2.46 × 10^–12^	− 0.09	7.16 × 10^–4^	2.77 × 10^–3^
SF3B1	0.09	7.83 × 10^–5^	4.84 × 10^–4^	0.07	1.78 × 10^–3^	3.76 × 10^–3^	0.10	8.48 × 10^–5^	5.26 × 10^–4^

*ARID1A, RUNX1, GATA3, TBX3, NF1, MAP2K4, PTEN, SMAD4, MAP3K1* and *SF3B1* significantly associated with all of the three clinical features. The column ‘CC’ (i.e., correlation coefficient denoted by *r*) measures the degree of association between the two variables: each driver gene versus each clinical feature. It takes on values ranging between − 1 and + 1. When *r* = 0, there is no relationship between the two variables. When *r* closer to 1, there is an increasingly strong positive (uphill) relationship between the two variables; otherwise, there is an increasingly strong negative (downhill) relationship between the two variables. CC: correlation coefficient.

### Co-expressed module-clinical feature association analysis using WGCNA

**Figure 3:**
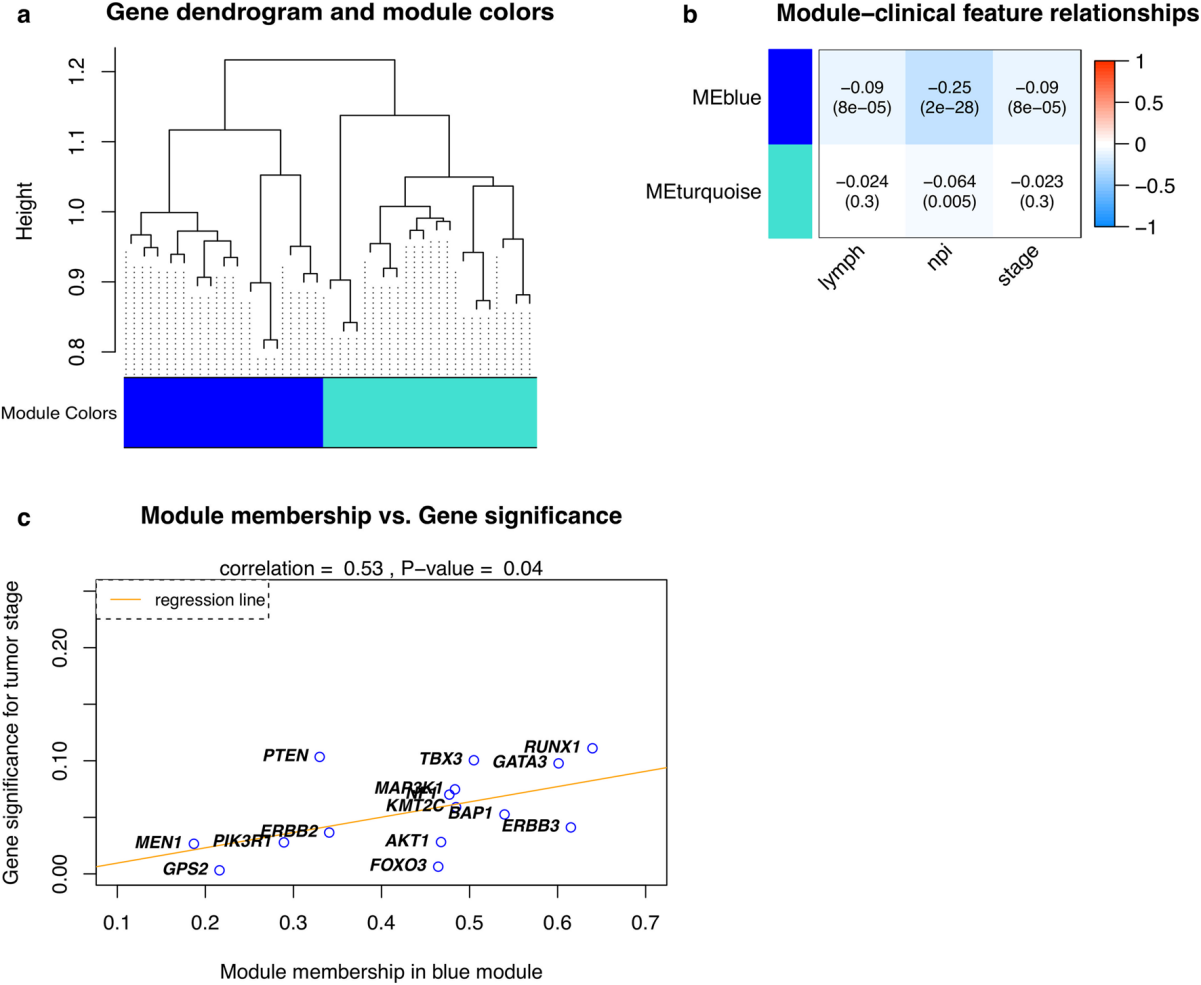
Co-expression network analysis for module-clinical feature associations. (**a**) Dendrogram of the identified driver genes on Topology Overlap Matrix-based dissimilarity**.** The dendrogram height corresponds to the coefficient of dissimilarity matrix that is for every pair of 31 driver genes, in which the low dissimilarities indicate two driver genes are close, whereas the high dissimilarities imply two driver genes are distant apart. Two co-expressed modules were detected and are shown in different colors. (**b**) Module–feature associations. Each row corresponds to a module eigengene (ME), column to a feature. Each cell contains the corresponding correlation coefficient and *P*-value. (**c**) A correlation between gene significance for tumor stage and module membership in the blue module. There is a significantly strong positive correlation between Gene significance and Module membership in this module (*r* = 0.58, *P*-value = 0.04). Significant genes (i.e., high Gene significance and high Module membership) in a single module are the ones having a significant association with a clinical feature considered. Abbreviation: lymph: number of positive lymph nodes, and npi: the Nottingham prognostic index, respectively.

Accordingly, Fig. 3A illustrates the dendrogram of the identified driver genes on their TOM-based dissimilarity (On top of Fig. 3a and Supplementary File 1, Figure S6). The height of the dendrogram indicates dissimilarity of two driver genes, in which low dissimilarities indicate that two driver genes are close (similar), whereas the high dissimilarities imply two driver genes are far apart (dissimilar). In addition, a total of two distinct gene co-expressed modules were found and represented in different colors, and they were arranged from large to small by the number of genes they included (i.e., 15 and 16 genes in the blue and turquoise modules, respectively).

Results of the module-clinical feature association analysis (Fig. 3b) indicated that the blue module was significantly negatively correlated (i.e., *r* < 0 and corresponding *P*-values ≤ 0.05) with all three clinical features (i.e., numbers of positive lymph nodes, the Nottingham prognostic index, and the tumor stages), whereas the turquoise module showed a significant negative correlation with the Nottingham prognostic index (i.e., *r* = 0.064 and corresponding *P*-value = 0.005). In addition, Fig. 3c shows that module membership and gene significance in the blue module for tumor stages are moderately correlated (i.e., *r* = 0.53 with *P*-value = 0.04). Also, *GATA3, ERRB3, RUNX1, BAP1,* and *TBX3* were the top five hub genes in the blue module, whereas in the turquoise module was *RB1, ZFP36L1, SMAD4, SF3B1,* and *CDKN1B*. *RUNX1, GATA3,* and *TBX3* were the top three significant and module memberships for tumor stages in the blue module. However, we realized that Table 5 and Fig. 3b show extremely modest effects with correlation coefficients close to zero. These results demonstrated that our identified driver genes are weakly correlated with selected clinical features.

### Patient stratification

**Figure 4:**
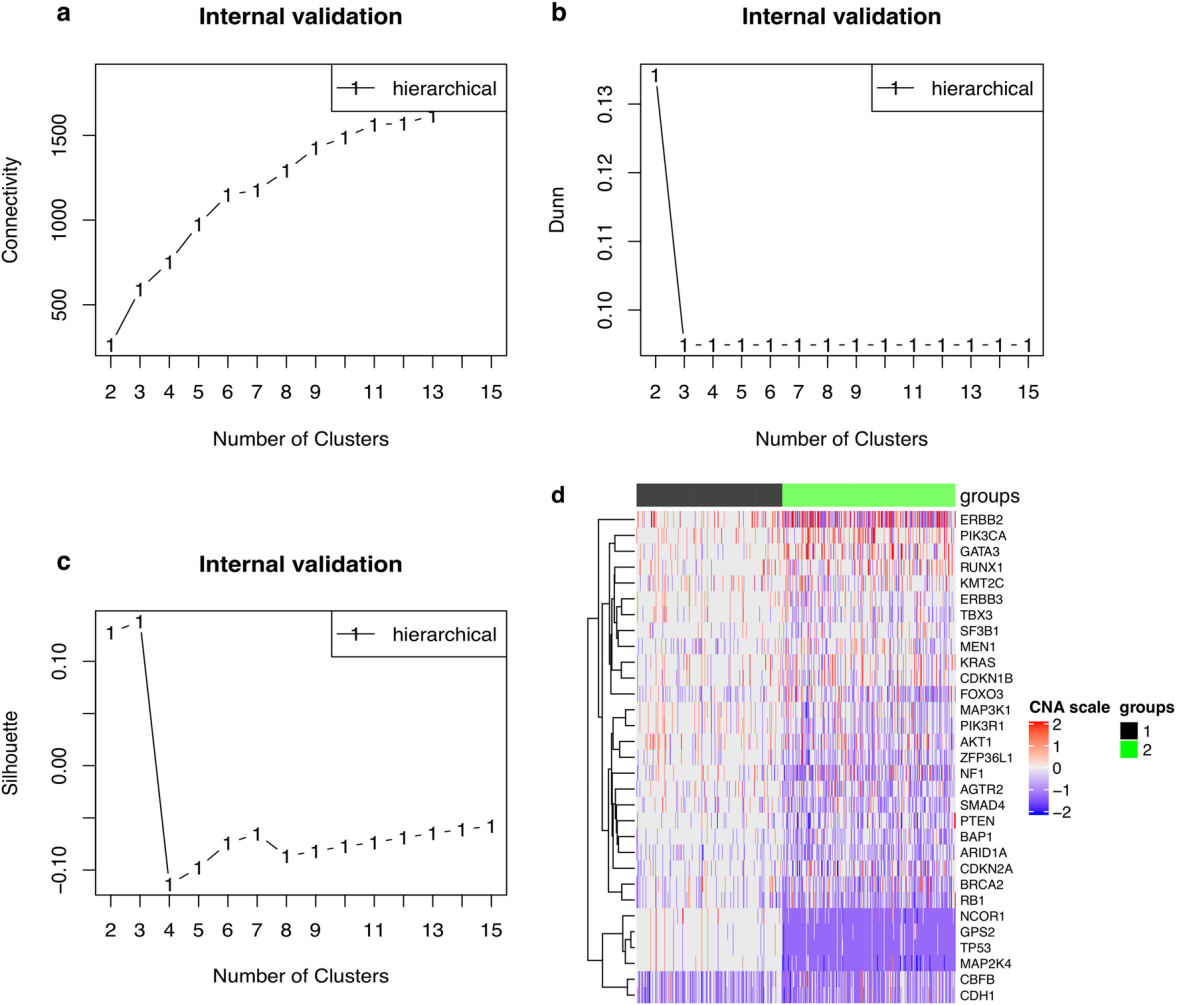
Optimal group number detection and difference in CNVs events between the identified groups. (**a**) Two optimal groups were determined by the connectivity. The connectivity computes the degree of connectedness of a given group partitioning. The connectivity shows the connectedness of a given cluster partitioning and has a value between 0 and infinity. The user should choose a point reaching the most minimized value (y-axis). (**b**) Two optimal groups were also determined by the Dunn’s index. The Dunn’s index (y-axis) has a value between zero (poorly clustered observations) and infinity (well clustered observations), and the place where the black line of Dunn’s index plot peaks at, which implies that that group number is optimal. (**c**) Three optimal groups were determined by the Silhouette width. The average Silhouette has a value between -1 (poorly clustered observations) and 1 (well clustered observations), and the place where the black line of the Silhouette plot peaks at, which implies that that group number is optimal. (**d**) The heatmap indicates the differences in CNV event distribution of two subgroups. The dark red, red, grey, blue and dark blue represent high-level amplification, amplification, copy‐neutral, deletion and high-level deletion, respectively.

Similar to identifying the above co-expressed modules, here we classified BRCA patients into different groups based on the CNV data of the identified driver genes. Firstly, the agglomerative clustering algorithm (i.e., the Ward algorithm) was used to cluster into patient dendrogram hierarchically. Then, an optimal number of groups was determined by the connectivity (Fig. 4a), the Dunn’s index (Fig. 4b) and the average Silhouette width (Fig. 4c) and the BRCA patients were distributed to each group. Collectively, Fig. 4A–C show that two optimal number of groups for the 2,173 BRCA patients were identified, which implied that clustering the patients into two subgroups was the best solution. Finally, we visualized the given result using a heatmap plot implemented in the function ‘Heatmap’ of the R package, ComplexHeatmap^79^. The heatmap reports the differences in CNV events between the two patient groups (group 1: 993 patients and group 2: 1180 patients; Fig. 4d).

**Figure 5:**
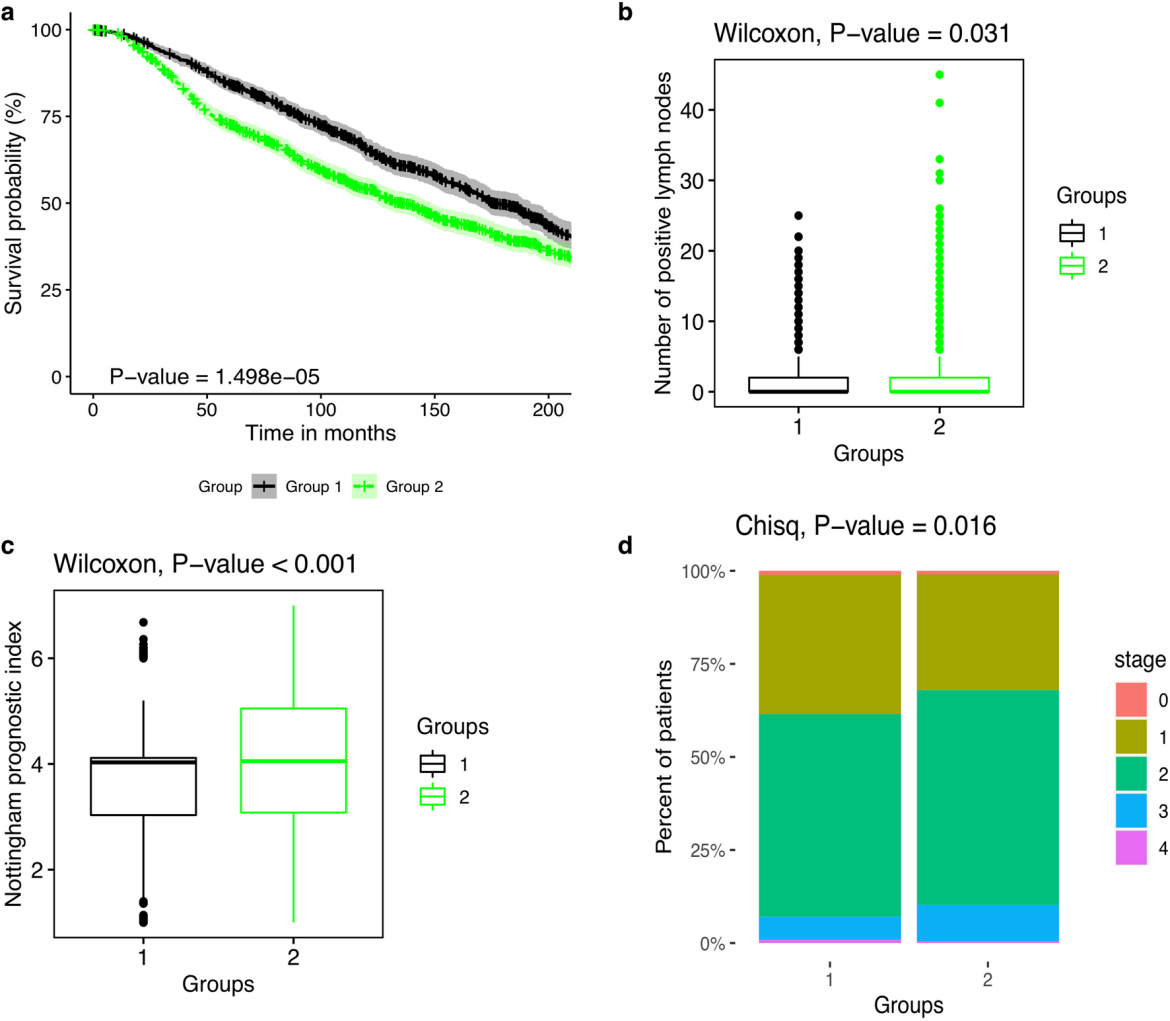
Differences between groups of BRCA patients in terms of clinical features. (**a**) survival rates, (**b**) the number of positive lymph nodes, (**c**) the Nottingham prognostic index and (d) cancer stage. Abbreviation: Chisq, Pearson’s *χ*^2^ test.

Particularly, the tumors in the second group exhibit significantly worse outcomes (HR is 1.29 with 95% CI (1.15–1.45), *P*-value < 0.01; group 1 is the reference) (Fig. 5a), higher numbers of positive lymph nodes (*P*-value = 0.02, Wilcoxon rank-sum test) (Fig. 5b; Supplementary File 2, Table S13), higher Nottingham prognostic index (*P*-value < 0.01, Wilcoxon rank-sum test) (Fig. 5c; Supplementary File 2, Table S13) and more advanced tumor stages than those in the first group (*P*-value < 0.01, Pearson’s *χ*^2^ test) (Fig. 5d; Supplementary File 2, Table S13).

## Discussions and conclusion

In this study, the two driver gene identification tools were used to detect the 35 driver genes in 2,369 BRCA samples, in which it showed that the 31 genes overlap with previously published common BRCA driver genes, whereas, to the best of our knowledge, the four leftover genes are yet-to-be-discovered BRCA drivers. The two OncodriveFML and OncodriveCLUSTL tools detected BRCA-related driver genes that are well established in other cancer types, such as *KRAS, ARID1A, CDKN2A, MEN1, BAP1, SMAD4*. This implies that therapies used in other clinical settings could be appropriate for BRCA with mutations in these genes^13^. Then, collectively, the five genes show a significant association with survival rate, whereas the ten genes are significantly but weakly correlated with all the three remaining clinical features through analyses between the expression levels of individual driver genes and clinical features. Nevertheless, this result is understandable since it is believed that genes do not function separately but work in concert to affect human health jointly. Indeed, recent studies have shown that individual genes averagely interact with at least four other genes^80^ and are relevant to 10 biological functions^81^. Cancer is a complex human disease caused by multiple molecular mechanisms, so gene co-expression networks are a potential approach to detecting a set of cancer-related genes that may be targeted for therapeutic interventions^82,83^ as well as the identification of hub genes that serve as fundamental roles in cancer. From such, we continue to implement the WGCNA tool to construct weighted driver gene co-expression networks. As a result, two co-expressed modules are detected; among them, one module is significantly negatively associated with the numbers of positive lymph nodes, the Nottingham prognostic index, and the tumor stage, whereas the other is significantly negatively associated with the Nottingham prognostic index. The top five hub genes are correspondingly identified in the two modules, indicating possession of a vast range of interactions with other genes and playing crucial roles in the co-expression network of those genes. Finally, hierarchical clustering analysis of all of the identified driver genes reveals two subgroups of BRCA patients. Further mining the heatmap, we realize that the mutation frequencies of all the driver genes are disparate across the groups: minimal frequencies in the first group and substantial frequencies in the second group. In addition, the second group is significantly poorer than the first group with regard to the three clinical features and patient survival. Therefore, more intensive treatment and frequent follow-up may be necessary for those patients assigned to the second group.

For our work, Fig. 1 describes an improved pipeline to perform the four most widely used analyses developed collectively based on prior works^7–15^, each of which could be highly adjustable. We envision this pipeline as a general but unlimited solution for cancer researchers wishing to deal with driver genes integrating multi-omics data for which parts of the proposed protocol can permit users to explore any other implementation suitable for their research. At Steps 2 and 4, the workflow implements the g:Profiler and WGCNA tools with basic settings, consistent with the implementation of previous studies. Users totally may refer to ref.^40^ and ref.^84^ to perform intensive analyses with them, respectively; however, it may lead to a considerable increase in computation time. For WGCNA, users must face a question: should one choose a ‘signed’ or ‘unsigned’ network? This selection affects how WGCNA treats the correlation of driver genes, in which ‘signed’ considers negative correlation coefficients of pairs of nodes (i.e., pairs of driver genes) to be unconnected, whereas ‘unsigned’ treats positive and negative correlations equally. There is no right answer existing, and previous works could make an inconsistent decision with one another. To avoid any confusion and follow the suggestion of WGCNA^19^, this study currently recommends a ‘signed’ network. At Step 3, for survival analysis, there are five survival types: overall survival, disease-specific survival, disease-free survival, recurrence-free survival, and distant metastasis-free survival that users can perform with single (i.e., univariate analysis) or combined (i.e., multivariate analysis) clinical features of interest^10–13,85^ to assess the association between individual driver genes and survival rates. At Steps 3 and 4, the work issues the main focus on the associations between clinical features and changed expression levels of identified driver genes. Based on the belief that gene expression is considered the first level of phenotype affected by the mutation/change on the gene, it is reasonable to investigate how the mutation affects its phenotype^7,8,10,12,15,49^. For example, a large number of non-coding drivers can regulate the expression of genes and driver genes^86,87^. However, it is optional, and you can use other omics data types for this step. At Step 5, in the patient stratification process, to limit computational burden, one prior study only selects 20 driver genes with the most frequent changes as input, including the ten most frequently amplified and the ten most frequently deleted driver genes^11^. Nevertheless, for methylation data, there is alternative to choose driver genes for this step^9,12^. In this study, we even use all the detected driver genes as input as already mentioned in the reference paper^13^. Finally, at Steps 3 and 5, in most cases, the distribution of omics data is skewed, so it is recommended that users should priorly select non-parametric methods for testing, such as preferring the Spearman’s rank correlation to the Pearson’s correlation. However, there may be applications in which parametric statistical methods are preferable.

Notably, our pipeline is considered as an improved and refined solution to those mentioned in previous studies. We attempt to make the analysis pipeline for the identification and characterization of driver genes more consistent and reproducible than old investigations. Also, most current driver identification tools are developed to detect genes with coding mutation, whereas the number of non-coding driver identification methods is considerably limited. However, an enormous number of mutations exist in non-coding regions (due to only around 2% of the human genome comprising of protein-coding regions); meanwhile, many previous studies^7,8,11^ selected coding-driver-specific tools such as OncodriveFM^21^. In this improved pipeline, we priorly select the two tools OncodriveFML and OncodriveCLUSTL that are two minor cases can identify both non-coding driver genes and infrequently mutated genes in the hope of encouraging researchers interested in this field to take this challenge into account when building a new tool or conducting a driver-gene-related study. For future work, the only way to validate non-coding cancer drivers is to do the literature review manually^86,88^ and most of the available databases used to validate only coding drivers; therefore, new resources for non-coding drivers should be built as rapidly as possible.

Last, the following limitations are essential to consider before performing the pipeline. Besides, we also make them feasible with the solutions attached. Firstly, cancer is a common disease, and most driver genes are now known, such as breast cancer^14^. Therefore, we suggest that when applying the proposed scheme for any cancer, we may skip Stage 1 (Fig. 1) and go directly to Stage 2 (Fig. 1) with well-established genes in that cancer type. In contrast, users should maintain Stage 1 to predict new drivers. A hint for the latter case is a combining approach using many driver identification tools simultaneously, for example, as seen in ref.^11^. Secondly, users can encounter several other limitations with Step 2 (Fig. 1, Stage 2) using g:Profiler that can see ref.^40^. Thirdly, the work proposes the computeC tool at Step 3 (Fig. 1, Stage 2) to perform correlation analysis using simple methods, but not more sophisticated methods for gene expression data like the R package ‘limma’^89^, etc. In the future, we will take this issue into account to improve the tool. Finally, a potential restriction when performing survival analysis at Steps 3 and 5 is that the pipeline deals with the censored data in a simple way. More specifically, for missing information, the function ‘coxph’ ignores it automatically, whereas, for end-of-study and loss-to-follow-up censoring, we select the approach of analyzing dichotomized data (see detailed implementations at Supplementary File 1). Consequently, these processes may pose problems to the analysis. Our solution is making assumptions about censoring to selecting the most appropriate statistical methods. For example, if the clinical data whose missing information is limited, the user can remove it; conversely, imputation methods should be taken into consideration.

In summary, we proposed an improved pipeline integrating state-of-the-art computational tools to identify and characterize the driver genes more efficiently and refinedly. Through the successful use of the proposed pipeline, many exciting results were identified, from revealing the four new driver genes, then discovering potential druggable targets as well as the two co-expressed modules, to detecting the two prognostic groups of BRCA patients. Obviously, it is valuable to develop individualized treatments for patients with BRCA in the future. Furthermore, we believe that this success, plus accompanying public codes, demonstrate the efficacy of the work as well as persuade other researchers to use the pipeline.

## Supplementary information

Supplementary information.

Supplementary information.

## Data Availability

The raw data used in the study are available in the cBioportal website: (https://www.cbioportal.org/study/summary?id=brca_tcga_pub). Approval by a local ethics committee was not required, and all the data can be immediately downloaded from the cBioportal website. R packages of computeC and geneSA are available on GitHub (https://github.com/huynguyen250896/computeC and https://github.com/huynguyen250896/geneSA), respectively.
