# Supplementary material for: Improving existing analysis pipeline to identify and analyze cancer driver genes using multi-omics data
Source: Sci Rep. 2020 Nov 25;10:20521. doi: 10.1038/s41598-020-77318-1 (PMC7688645; doi:10.1038/s41598-020-77318-1)
Supplement: Supplementary file 1 — Supplementary information. [file 41598_2020_77318_MOESM1_ESM.pdf]

Improving existing analysis pipeline to identify and analyze cancer driver genes using multi-omics data

Quang-Huy Nguyen[1,2] and Duc-Hau Le[1,3,*]
[1]Department of Computational Biomedicine, Vingroup Big Data Institute, Hanoi, Vietnam.
[2]Faculty of Pharmacy, Dainam University, Hanoi, Vietnam.
[3]School of Computer Science and Engineering, Thuyloi University, Hanoi, Vietnam.

Supplementary Information

User manual

Table of Contents

1. Introduction..3

2. Dataset..3

3. Identification of driver genes using OncodriveFML and OncodriveCLUSTL (Fig.1, Stage 1; Step 1)..4

3.1. Identification of driver genes using OncodriveFML..4

3.2. Identification of driver genes using OncodriveCLUSTL...................................5

4. Enrichment Analysis using g:Profiler (Fig.1, Stage 2; Step 2)..........................6

5. Individual gene-clinical feature association analysis (Fig.1, Stage 2; Step 3).................7

5.1. Association between individual driver genes and survival rate........................7

5.2. Identify which driver genes are significantly correlated with other clinical features 8

6. Co-expressed module-clinical feature association analysis using WGCNA (Fig.1, Stage 2; Step 4)..9

6.1. Weighted co-expression driver gene network construction............................10

6.2. Detect associations of functional modules to clinical features, identification of significant genes and hub genes in each of those modules...13

7. Patient stratification (Fig.1, Stage 2; Step 5)..16

7.1. Pre-processing clinical data & CNVs data..16

7.2. Patient stratification by the identified driver genes...17

7.3. Comparision between the identified patient groups..20

8. References..25

1. Introduction

The cumulative of genes carrying mutations is vital for the establishment and development of cancer. However, this line of driver gene exploring research has selected and used types of tools and models of analysis unsystematically and discretely. Also, the previous studies may have neglected low-frequency drivers and seldom predicted subgroup specificities of identified driver genes. In this document, we propose an improved driver gene identification and analysis pipeline integrating state-of-the-art tools to solve the above challenges (Fig.S1).

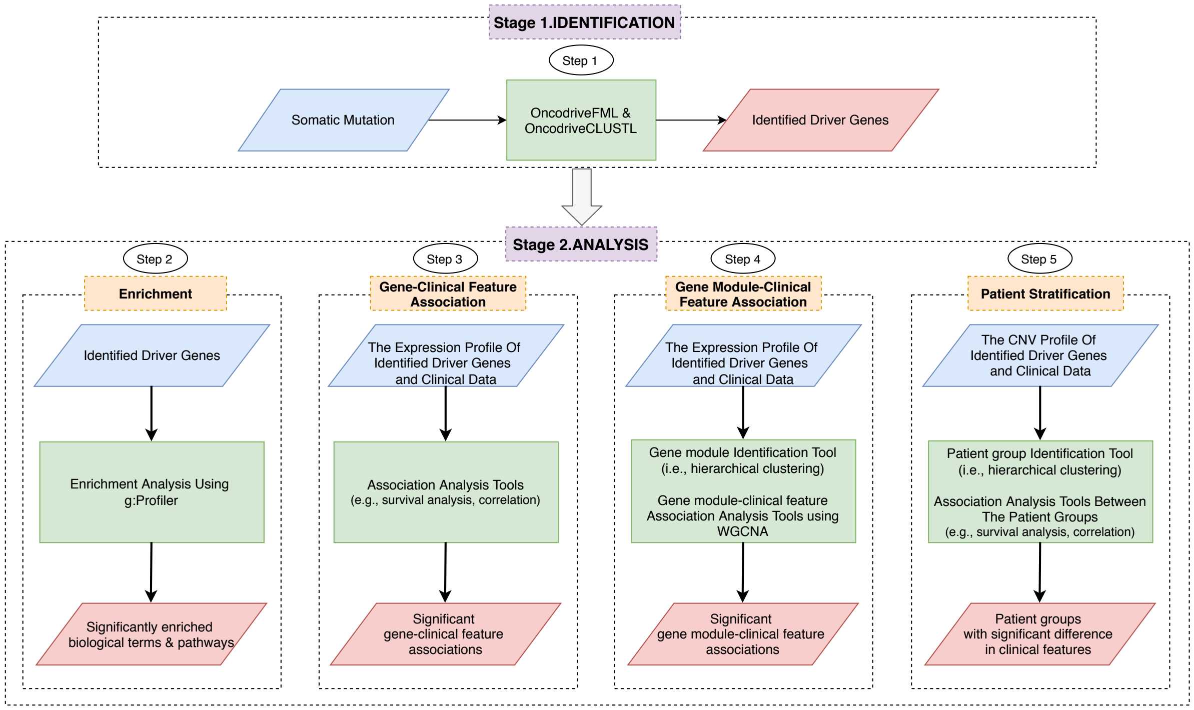

Figure S1. Improved analysis pipeline for identification and analysis of driver genes. The scheme comprises two stages: identification and analysis, in which the former uses the OncodriveFML and OncodriveCLUSTL to identify driver genes with somatic mutation data as input, and the latter performs the four most widely focused analyses to deal with those driver genes. Abbreviation: CNV, Copy number variations.

Here, we use the METABRIC breast cancer (BRCA) dataset [1] as an example to demonstrate the use of our work to (Stage 1) identify and (Stage 2) analyze the driver genes.

2. Dataset

The breast cancer data are downloaded from the cBioPortal for Cancer Genomics (http://www.cbioportal.org) [2]. It contains METABRIC breast cancer cohort assembled from 2,509 primary breast cancer patients with 548 matched normals in the United Kingdom and Canada [3]. The gene expression microarray data are generated using the Illumina Human v3 microarray for 1,904 samples, while the CNVs data are measured on the Affymetrix SNP 6.0 platform for 2,173 samples, and 17,272 somatic mutations of 173 genes for 2,369 samples are detected on the Illumina HiSeq 2,000 platform.

Each data type is a tabular file with a header that must contain, at least, the following columns:

File	Data type	Format (Data fields)
data_mutations_extended.txt	Mutation	- For OncodriveFML: http://bbglab.irbbarcelona.org/oncodrivefml/faq#qfilesformat<br>- For OncodriveCLUSTL: http://bbglab.irbbarcelona.org/oncodriveclustl/faq#qmutfileformat
data_mRNA_median_Zscores.txt	Gene expression	- Hugo_Symbol: gene names<br>- The remaining columns are BRCA patients
data_CNA.txt	Copy number aberration	- Hugo_Symbol: gene names<br>- The remaining columns are BRCA patients
data_clinical_patient.txt	Clinical feature (traits)	1. PATIENT_ID<br>2.LYMPH_NODES_EXAMINED_POSITIVE: Number of positive lymph nodes<br>3. stage: Tumor stages<br>5. NPI: Nottingham prognostic index<br>6. OS_MONTHS: Follow-up months<br>7. OS_STATUS: BRCA patients' status

Table 1. The description of data used in the study. The format of all data types used in the study.

3. Identification of driver genes using OncodriveFML and OncodriveCLUSTL (Fig.1, Stage 1; Step 1)

Identification of driver genes in BRCA is implemented using the two tools OncodriveCLUSTL 1.1 [4] and OncodriveFML 1.0 [5]. They are available as a friendly web-based application at http://bbglab.irbbarcelona.org/oncodriveclustl/analysis and http://bbglab.irbbarcelona.org/oncodrivefml/analysis#, respectively. Both of them require a specific format data that can refer to the heading **?FAQ** at each below the corresponding website. In general, the requirement is simple, and the somatic mutation data from popular omics data sources (e.g., METABRIC and TCGA) meets it at the origin format; therefore, directly upload the file *data_mutations_extended.txt* to each tool.

3.1. Identification of driver genes using OncodriveFML

The parameters of the OncodriveFML tool are set to default values with the exception of the sequencing parameter (targeted sequencing) and the scoring system CADD v1.3 [6] (the latest version at the time of this writing).

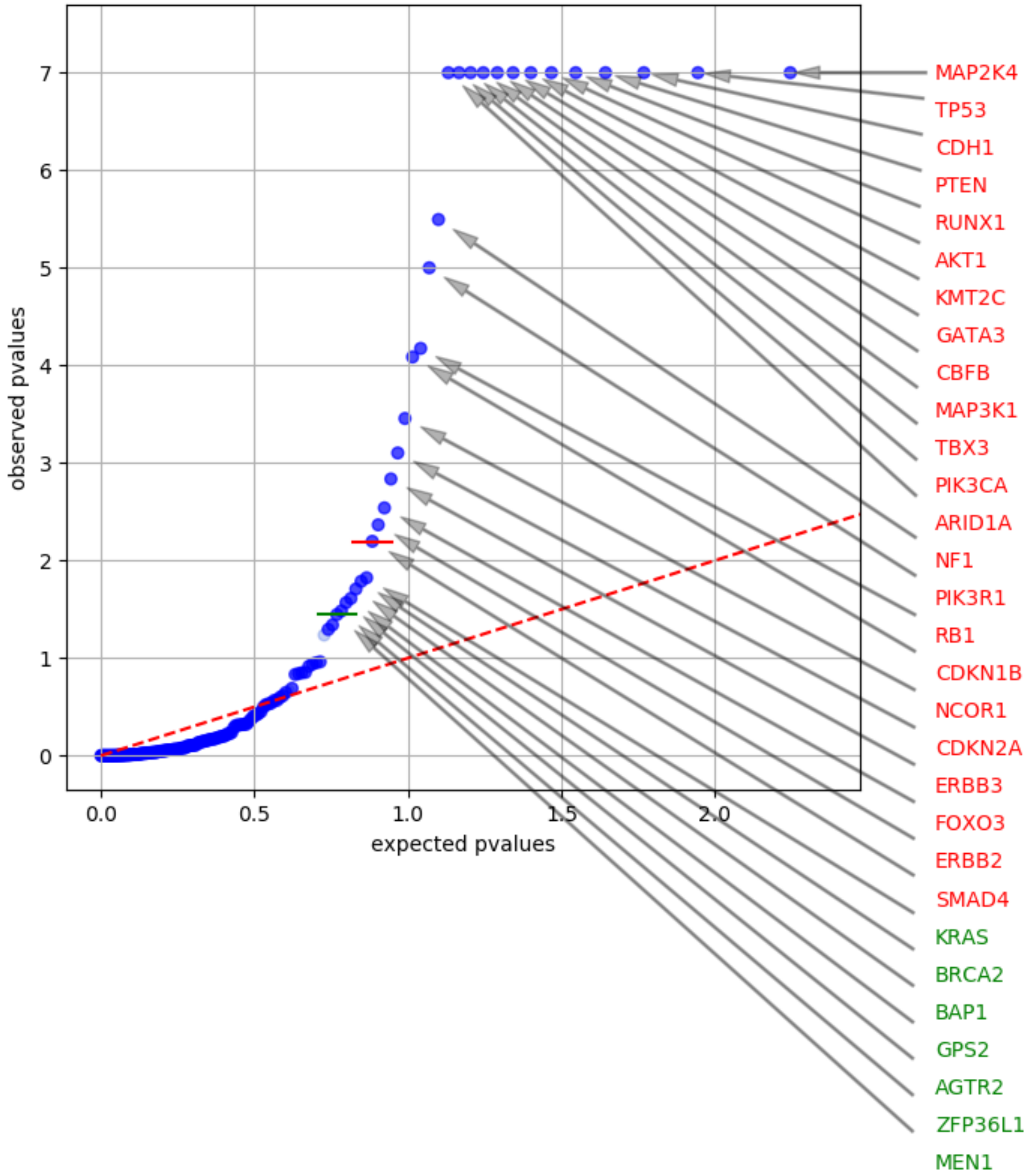

Figure S2. OncodriveFML result. List of driver genes is predicted by OncodriveFML from the analysis data. A comprehensive list of driver genes can be found from the downloaded tsv file, *<day of the file created>_input-oncodrivefml.tsv*. Red gene names indicate driver genes with Q-value < 0.1 (Benjamini-Hochberg procedure), and green gene names indicate driver genes with Q-value < 0.25. X and Y-axis mean negative log10-transformation of P-value.

3.2. Identification of driver genes using OncodriveCLUSTL

The parameters of the OncodriveCLUSTL tool are set to default values with the exception of the selection of 'concatenate' option.

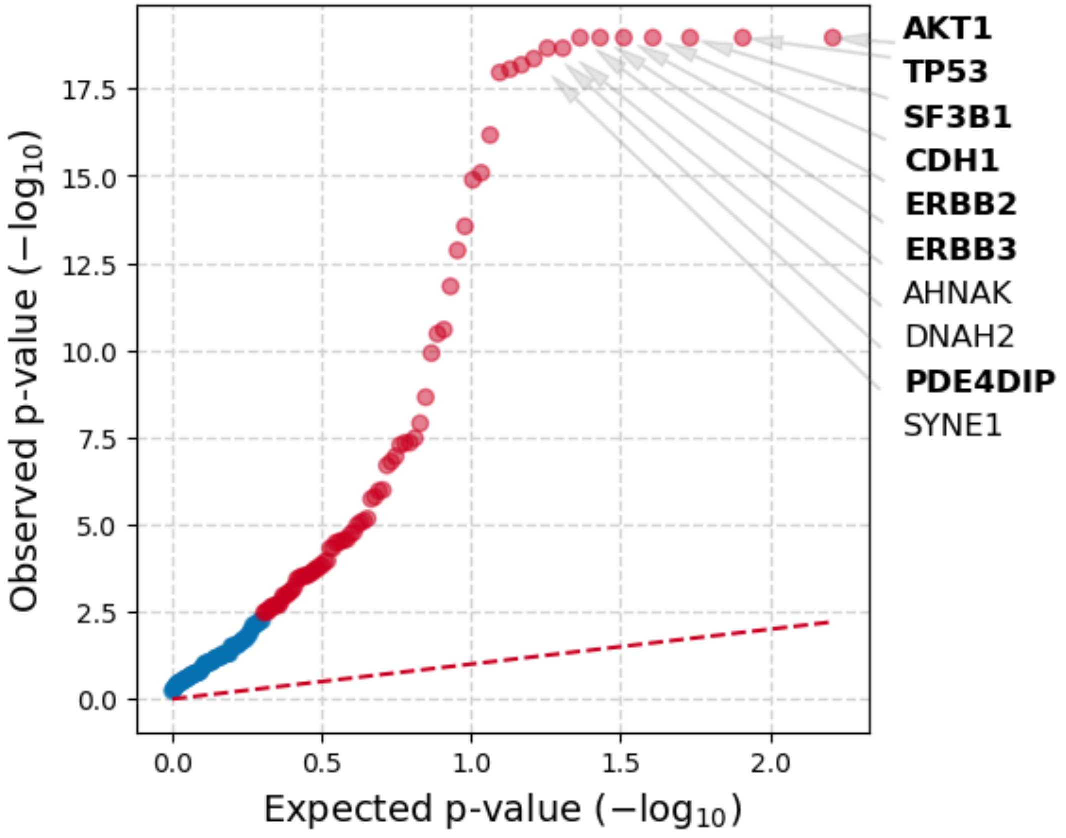

Figure S3. OncodriveCLUSTL result. List of driver genes is predicted by OncodriveCLUSTL. A comprehensive list of driver genes can be found from the downloaded compressed zip file, *<day of the file created>_compressed_file.zip*. Gene names predicted to be driver genes if Q-value < 0.01 (Benjamini-Hochberg procedure). Names in bold denote genes annotated in the Cancer Gene Census (CGC) database.

As a result, collectively combining two sets of driver genes from the above figures, a total of 35 unique driver genes are detected by the two tools (Supplementary File 2, Table S1). Through the validation process, 31 *bona fide* driver genes are reserved for downstream analyses.

4. Enrichment Analysis using g:Profiler (Fig.1, Stage 2; Step 2)

The study identifies significantly enriched GO biological processes and KEGG pathways using g:Profiler to characterize the functional enrichment of all driver genes. Specific procedures are shown as follows:
Firstly, users can open the g:Profiler website at https://biit.cs.ut.ee/gprofiler/gost. Input is the 31 identified driver genes (Supplementary File 2, Table S1). In 'Data sources' tab, we only choose 'GO biological process' option under the sub-tab 'Gene Ontology' and 'KEGG' option under the sub-tab 'biological pathways'. The remaining options are excluded or kept as they are (i.e., default). Finally, click 'Run query' to get the results. A full list of enriched GO terms and pathways can be downloaded by clicking the button 'CSV' in the 'Detailed Results' tab. The downloaded result is a csv file, *gProfiler_<organism>_<day of the file created>_<time of day of the file created>__intersections.csv.*

As a result, g:Profiler yields 483 significantly enriched biological processes (Supplementary File 2, Table S4) and 71 significantly enriched KEGG pathways (Supplementary File 2, Table S5).

Because g:Profiler is an up-to-date gene enrichment analysis tool, the results can not be reproduced if using in a different version. The version of g:Profiler in this work is e100_eg47_p14_7733820. Users can find the older versions from https://biit.cs.ut.ee/gprofiler/archives/.

5. Individual gene-clinical feature association analysis (Fig.1, Stage 2; Step 3)

Firstly, we must setup and load some essential libraries and data objects. Then, we pre-process gene expression data & clinical data. Specifically, we only retain 1,904 BRCA patients having both clinical and gene expression data for the 31 identified driver genes.

```
#Pre-processing gene expression data & clinical data
source("1.Clinical-preprocess.R")
```

5.1. Association between individual driver genes and survival rate

For step 3 (Fig. S1, stage 2), source the R.script (2.Clinical-SA.R) that contains:

- Prepare the data by creating event vector for median expression data, in which values above the median are assigned as up-regulated (= "up"), and values below the median are assigned as down-regulated (= "down"). This can be done using the function apply().
- The function 'geneSA' (https://github.com/huynguyen250896/geneSA) implements a log-rank test in univariate Cox regression analysis with a proportional hazards model to associate the expression of each driver gene with the overall survival of the patients automatically and separately. The function will report hazard ratios (column 'HR'), 95% confidence intervals (column 'confidence_intervals'), P-values, and Q-values of each driver gene.

```
#library
if(!require(devtools)) install.packages("devtools")
devtools::install_github("huynguyen250896/geneSA")
library(geneSA)
if(!require(rlist)) install.packages("https://cran.r-project.org/src/contrib/Archive/rlist/rlist_0.4.tar.gz", repos = NULL); library(rlist)

#check what driver genes are significantly correlated with survival rate
source("2.Clinical-SA.R")
```

Table 2 shows nine driver genes significantly correlated with survival rate.

Gene	HR (95% CI)	P-value	Q-value
AKT1	1.30 (1.15-1.46)	1.48×10^{-05}	2.29×10^{-04}
KMT2C	1.24 (1.10-1.40)	3.47×10^{-04}	2.69×10^{-03}
KRAS	1.20 (1.07-1.35)	2.30×10^{-03}	1.19×10^{-02}
PTEN	0.85 (0.76-0.96)	7.92×10^{-03}	2.73×10^{-02}
TBX3	0.84 (0.75-0.95)	4.91×10^{-03}	1.90×10^{-02}
PIK3R1	0.84 (0.75-0.95)	4.37×10^{-03}	1.93×10^{-02}
MAP3K1	0.82 (0.73-0.93)	1.23×10^{-03}	7.61×10^{-03}
SMAD4	0.78 (0.69-0.88)	4.85×10^{-05}	5.01×10^{-04}
MAP2K4	0.76 (0.67-0.85)	4.57×10^{-06}	1.42×10^{-05}

Table 2. Association between the expression of driver genes and the overall survival of BRCA patients. Three genes including *AKT1, KMT2C,* and *KRAS* with above-median expression level and six genes including *PIK3R1, PTEN, SMAD4, MAP3K1, MAP2K4* and *TBX3* with below-median expression level significantly associated with a shortened lifespan. HR is a measure that helps determine whether either of two expression levels of each driver gene will result in an increased (i.e., HR > 1) or decreased (i.e., HR < 1) probability of experiencing the defined event (i.e., death), at any time (below-median expression level is the reference). P-value is computed by the Cox proportional hazard method to test the statistical difference of the given results. Q-value is computed following the Benjamini-Hochberg procedure. HR: hazard ratio. 95% CI: 95% confidence interval.

Below are the results gained from the KMplot database:

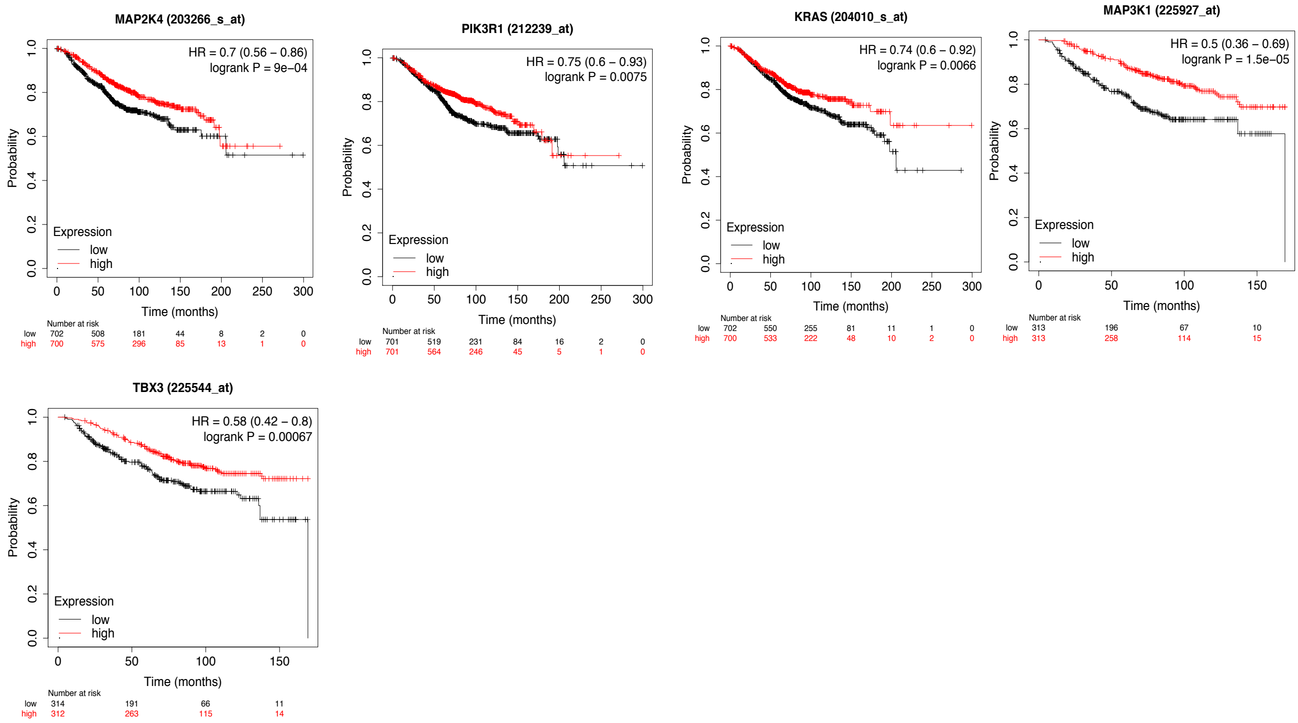

Figure S4. Kaplan-Mayer survival curves in the KMplot dataset. Five driver genes are predicted by the KMplot databse, including *MAP2K4, PIK3R1, KRAS, MAP3K1,* and *TBX3.*

5.2. Identify which driver genes are significantly correlated with other clinical features

We identify which driver genes are significantly correlated with other clinical features, including numbers of lymph nodes, the Nottingham prognostic index, and the pathologic stages. Specifically, 'computeC' (https://github.com/huynguyen250896/computeC) computes the correlation coefficients between individual drivers and clinical features based on Spearman's rank correlation (default value). The function will report correlation coefficients (column 'CC'), P-values, and Q-values of each driver gene with each clinical feature of interest.

```
#library
devtools::install_github("huynguyen250896/computeC")
library(computeC) #compute the correlation

#check what driver genes are significantly correlated with three other
clinical features
source("3.Clinical-corr.R")
```

Gene	Number of lymph nodes			Nottingham prognostic index			Cancer Stage		
	**CC**	**P-value**	**Q-value**	**CC**	**P-value**	**Q-value**	**CC**	**P-value**	**Q-value**
ARID1A	-0.06	0.01	0.02	-0.13	1.31×10^{-8}	3.20×10^{-8}	-0.10	1.13×10^{-4}	4.73×10^{-4}
RUNX1	-0.14	1.65×10^{-9}	3.63×10^{-8}	-0.25	2.20×10^{-16}	2.42×10^{-15}	-0.11	2.97×10^{-5}	3.12×10^{-4}
GATA3	-0.01	1.27×10^{-5}	9.31×10^{-5}	-0.28	2.20×10^{-16}	4.84×10^{-15}	-0.12	3.83×10^{-5}	2.68×10^{-4}
TBX3	-0.10	9.10×10^{-6}	1.00×10^{-4}	-0.18	1.44×10^{-15}	6.31×10^{-15}	-0.12	6.12×10^{-5}	3.21×10^{-4}
NF1	-0.09	5.77×10^{-5}	3.17×10^{-4}	-0.08	2.23×10^{-4}	3.07×10^{-4}	-0.07	6.36×10^{-3}	0.02
MAP2K4	-0.08	4.61×10^{-4}	1.69×10^{-3}	-0.22	2.20×10^{-16}	1.21×10^{-15}	-0.07	6.99×10^{-3}	0.02
PTEN	-0.08	6.02×10^{-4}	1.89×10^{-3}	-0.23	2.20×10^{-16}	1.61×10^{-15}	-0.11	2.93×10^{-5}	6.15×10^{-4}
SMAD4	-0.06	0.01	0.03	-0.10	1.79×10^{-5}	3.29×10^{-5}	-0.08	1.48×10^{-3}	3.89×10^{-3}
MAP3K1	-0.06	0.01	0.03	-0.16	6.35×10^{-13}	2.00×10^{-12}	-0.09	7.16×10^{-4}	2.15×10^{-3}
SF3B1	0.09	7.83×10^{-5}	1.02×10^{-3}	0.07	1.78×10^{-3}	3.31×10^{-3}	0.10	8.48×10^{-5}	1.19×10^{-3}

Table 3. Association between the expression of driver genes and the other clinical features. *ARID1A, RUNX1, GATA3, TBX3, NF1, MAP2K4, PTEN, SMAD4, MAP3K1* and *SF3B1* significantly associated with all of the three clinical features. The column 'CC' (i.e., correlation coefficient denoted by r) measures the degree of association between the two variables: each driver gene versus each clinical feature. It takes on values ranging between -1 and +1. When r = 0, there is no relationship between the two variables. When r closer to 1, there is an increasingly strong positive (uphill) relationship between the two variables, otherwise is an increasingly strong negative (downhill) relationship between the two variables. CC: correlation coefficient.

6. Co-expressed module-clinical feature association analysis using WGCNA (Fig.1, Stage 2; Step 4)

We first install all the required packages before the analysis and set up a basic setting following the WGCNA's recommendation.

```
#library
install.packages(c("dynamicTreeCut","flashClust","Hmisc","WGCNA"))
 if(!requireNamespace("BiocManager", quietly = TRUE))
install.packages("BiocManager")

BiocManager::install("preprocessCore")
library("dynamicTreeCut").   # Module identification
library("flashClust") # Fast implementation of hierarchical
clustering
library("Hmisc")   #perform variables clustering
library("WGCNA")    #WGCNA tool
library(purrr)      #data processing
library(cluster)     # compute agglomerative coefficient

# The following setting is important, do not omit.
options(stringsAsFactors = FALSE);
enableWGCNAThreads()# Allowing parallel execution
```

6.1. Weighted co-expression driver gene network construction.

The function 'pickSoftThreshold' helps to find the optimal soft threshold tomake the co-expression matrix of the identified drivers fit a scale-free topology model. The set of soft-thresholding powers is default following the recommendation of WGCNA's authors. As shown in Fig.S5, the work recommends choosing a point at which R^2 reaches the peak for the first time (y-axis), as described in the paper [7]. From that, we choose the soft-power $\beta = 6$.

```
#create  clinical_exp1 for this step
clinical_exp1 = clinical_exp[,-c(3,5:6)] #remove OS_MONTHS,
OS_STATUS and status columns

# Choose a set of soft-thresholding powers
powers = c(c(1:10), seq(from = 12, to=20,
by=2))#1,2,3,4,5,6,7,8,9,10,12,14,16,18,20
# Call the network topology analysis function
sft  =  pickSoftThreshold(t(exp_dri_norm),  powerVector  =  powers,
verbose = 5, networkType = "signed")
# Plot the results:
sizeGrWindow(9, 5)
cex1 = 0.9;
# Scale-free topology fit index as a function of the soft-
thresholding power
plot(sft$fitIndices[,1], -
sign(sft$fitIndices[,3])*sft$fitIndices[,2],
                    xlab="Soft Threshold (power)",ylab="Scale Free
Topology Model Fit,signed R^2",type="n",
                    main = paste("Scale independence"));
      text(sft$fitIndices[,1], -
sign(sft$fitIndices[,3])*sft$fitIndices[,2],
                    labels=powers,cex=cex1,col="red");
# this line corresponds to using an R^2 cut-off of h
abline(h=0.24,col="red")
```

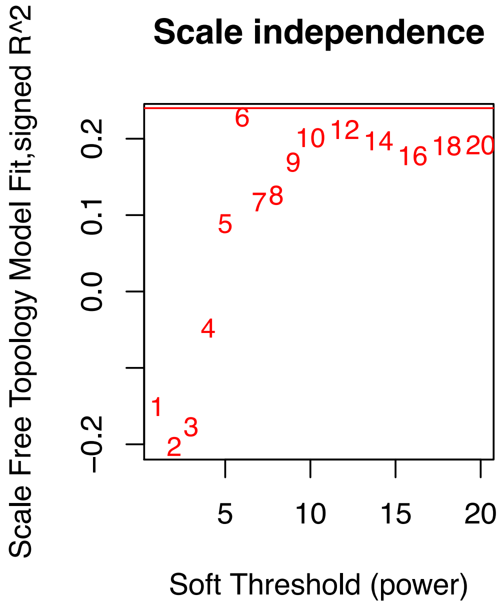

Figure S5 | Analysis of network topology for various soft-thresholding powers. The graph shows the scale-free fit index (y-axis) as a function of the soft-thresholding power β (x-axis).

We now calculate the adjacencies using the function 'adjacency' with identified soft-power β = 6, then turn the adjacency to Topological Overlap Matrix (TOM) with the function 'TOMsimilarity' before calculating the corresponding dissimilarity:

```
# Calculate the adjacencies
softPower = 6;
adjacency = adjacency(t(exp_dri_norm), power = softPower,
                      type = "signed");

# Turn adjacency into topological overlap
TOM = TOMsimilarity(adjacency, TOMType = "signed");
dissTOM = 1-TOM
```

The functions 'pickSoftThreshold', 'adjacency', and 'TOMsimilarity' use respectively the arguments 'networkType', 'type', and 'TOMType' to allow the user to construct a signed or unsigned network.

Next, detect the co-expressed modules and plot them:
(i) Creation of a gene tree

- Perform agglomerative hierarchical clustering using the 'agnes' function to get the agglomerative coefficient. As a result, Ward's method (i.e., ward.D2) is the best solution.
- The function 'hclust' creates a gene tree (Fig.S6 and the top side of Fig.S7) by combining a dissimilarity matrix of all the drivers, calculated based on Pearson's correlation, with Ward's method, finding the relationship between the driver genes.

(ii) Identification of co-expressed modules based on the gene tree.

- The function 'cutreeDynamic' for the dynamicTreeCut package distributes the identified driver genes to each resulting module

(iii) The function 'plotDendroAndColors' visualizes the identified co-expressed modules which are colored separately (Fig.S6).

```
# methods to assess
m <- c( "average", "single", "complete", "ward")
names(m) <- c( "average", "single", "complete", "ward")

# function to compute agglomerative coefficient
set.seed(2583)
ac <- function(x) {
  agnes(exp_dri_norm, method = x)$ac
}
map_dbl(m, ac) # Agglomerative coefficient of each agglomeration
method

#assign gene names from adjacency to dissTOM
rownames(dissTOM) = rownames(adjacency)
colnames(dissTOM) = colnames(adjacency)
# Call the hierarchical clustering function
geneTree = hclust(as.dist(dissTOM), method = "ward.D2");
# Plot the resulting clustering tree (dendrogram)
sizeGrWindow(12,9)
```

```
plot(geneTree, xlab="", sub="", main = "Gene clustering on TOM-based
dissimilarity",cex.lab = 1,cex.axis = 1, cex.main = 1.2);
```

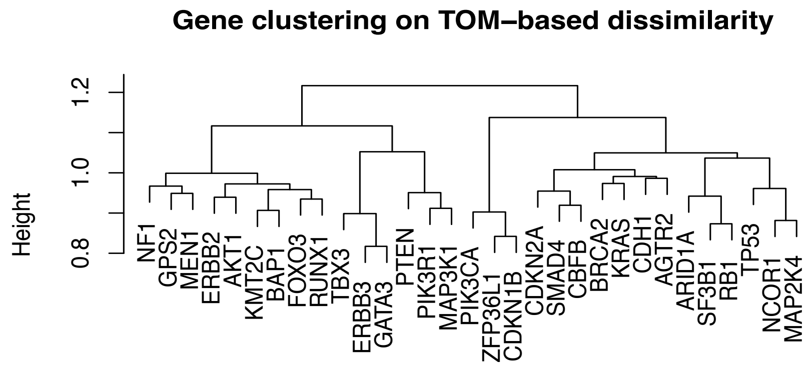

Figure S6 | Gene tree. Clustering dendrogram of genes, with dissimilarity based on topological overlap, together with assigned module colors.

```
#We set the minimum module size at 10:
minModuleSize = 10;
# Module identification using dynamic tree cut:
dynamicMods = cutreeDynamic(dendro = geneTree, distM = dissTOM,
                            minClusterSize = minModuleSize);
table(dynamicMods)
# dynamicMods
# 1  2
# 16 15
```

Argument 'minClusterSize' designates the minimum number of driver genes appearing in each identified module. At best, according to prior studies, users should consider choosing 10 for reproducibility. The other ones should be left at default.

```
# Convert numeric lables into colors
moduleColors  = labels2colors(moduleColors )
# Plot the dendrogram and colors underneath
sizeGrWindow(5,6)
plotDendroAndColors(geneTree, moduleColors, "Module Colors",
                    dendroLabels = FALSE, hang = 0.03,
                    addGuide = TRUE, guideHang = 0.05,
                    main = "Gene dendrogram and module colors")
```

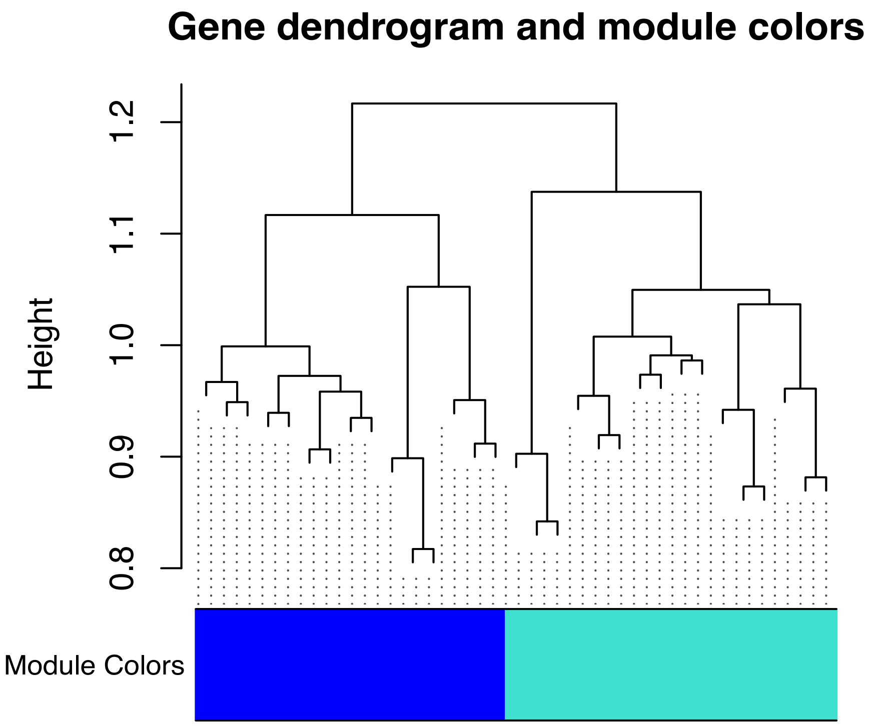

Figure S7 | Gene dendrogram and module colors. Clustering dendrogram of genes, with dissimilarity based on the topological overlap, together with assigned module colors.

6.2. Detect associations of functional modules to clinical features, identification of significant genes and hub genes in each of those modules.

The function 'labeledHeatmap' further analyzes and visualizes the associations between the identified modules and clinical features. The resulting color-coded table is shown in Fig. S8.

```
#Define numbers of genes and samples
nGenes = ncol(t(exp_dri_norm));
nSamples = nrow(t(exp_dri_norm));
# Recalculate MEs with color labels
MEs0 = moduleEigengenes(t(exp_dri_norm), moduleColors)$eigengenes
MEs = orderMEs(MEs0)
moduleTraitCor = cor(MEs, clinical_exp1, use = "p");
moduleTraitPvalue = corPvalueStudent(moduleTraitCor, nSamples);

sizeGrWindow(20,6)
# Will display correlations and their p-values
textMatrix = paste(signif(moduleTraitCor, 2), "\n(",
                   signif(moduleTraitPvalue, 1), ")", sep = "");
dim(textMatrix) = dim(moduleTraitCor)
par(mar = c(6, 8.5, 3, 3));

# Display the correlation values within a heatmap plot
labeledHeatmap(Matrix = moduleTraitCor,
               xLabels = names(clinical_exp1),
               yLabels = names(MEs),
               ySymbols = names(MEs),
```

```
colorLabels = FALSE,
colors = blueWhiteRed(50),
textMatrix = textMatrix,
setStdMargins = FALSE,
cex.text = 0.8,
zlim = c(-1,1),
main = paste("Module-clinical feature relationships"))
```

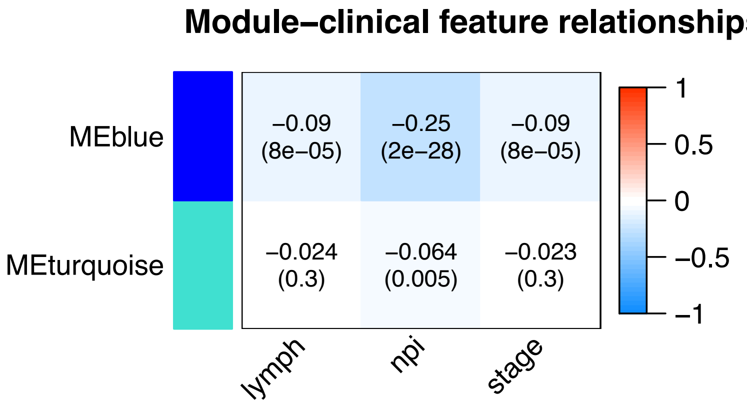

Figure S8 | Co-expressed module–feature associations. Each row corresponds to a module, column to a clinical feature. Each cell contains the correlation coefficient and its corresponding p-value. Abbreviation: lymph, numbers of lymph nodes, npi, Nottingham prognostic index, stage, tumor stages.

We discover significant genes (i.e., high Gene significance and high Module membership, Fig.S9) in a single module having a significant association with clinical features (the blue module is an example, the others can be done similarly)

```
####blue
# names (colors) of the modules
# Define variable stage containing the stage column of exp_dri_norm
stage = as.data.frame(clinical_exp1$stage);
names(stage) = "stage"
# names (colors) of the modules
modNames = substring(names(MEs), 3)
geneModuleMembership = as.data.frame(cor(t(exp_dri_norm), MEs, use =
"p"));
MMPvalue=as.data.frame(corPvalueStudent(as.matrix(geneModuleMembersh
ip), nSamples));
names(geneModuleMembership) = paste("MM", modNames, sep="");
names(MMPvalue) = paste("p.MM", modNames, sep="");
geneTraitSignificance=as.data.frame(cor(t(exp_dri_norm), stage, use
= "p"));
GSPvalue=as.data.frame(corPvalueStudent(as.matrix(geneTraitSignifica
nce), nSamples));
names(geneTraitSignificance) = paste("GS.", names(stage), sep="");
names(GSPvalue) = paste("p.GS.", names(stage), sep="");
```

```
#Intramodular analysis: identifying genes with high GS and MM
module = "blue"
column = match(module, modNames);
moduleGenes = moduleColors==module;
sizeGrWindow(7, 7);
par(mfrow = c(1,1));
x <- abs(geneModuleMembership[moduleGenes, column])
y <- abs(geneTraitSignificance[moduleGenes, 1])
limit <- range(c(x,y))
r <- round(cor(x,y),2)
plot(    x,

         y,

         ylim =range(0.00,0.25), xlim =range(0.1,0.7),

         xlab =paste("Module Membership in", module, "module"),

         ylab ="Gene significance for stage", col = module)

fit <-lm(y~x)
pintra= round(summary(fit)$coefficients[,4][[2]],2) #p-value
abline(fit, col='orange')
legend('topleft', col = "orange", lty = 1, box.lty =
1,legend='regression line', cex= 0.8)
mtext(paste('correlation = ', r, ",", "P-value = ", pintra))
title(paste("Module membership vs. gene significance\n"))
text(y~x,labels=rownames(exp_dri_norm)[moduleColors=="blue"],data=ex
p_dri_norm, cex=0.8, font=4, pos = 3) #add label
```

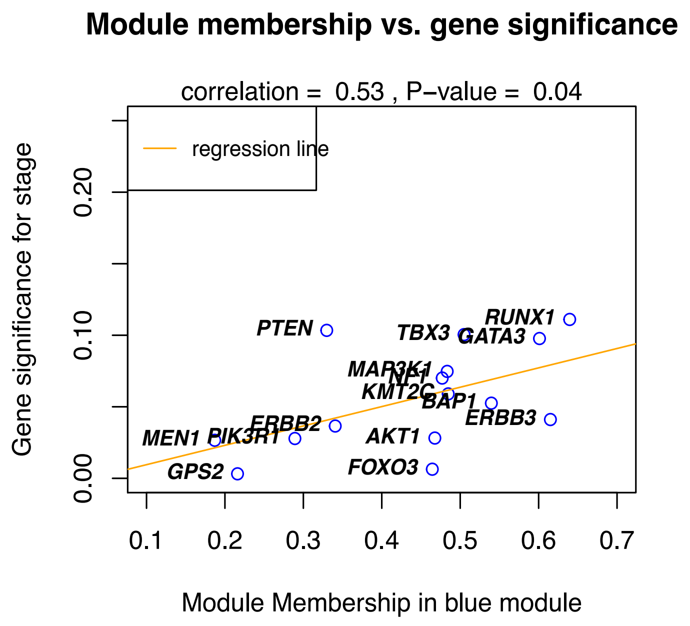

Figure S9 | Module membership and gene significance. A correlation of the blue module to the tumor stages with all genes in it.

Alternatively, the function `verboseScatterplot` provided in the package WGCNA can generate the similar result in Fig.S9 in a more easier way, but users can not draw a regression line (orange) and add gene names next to the corresponding blue circles:

```
#Alternatively
verboseScatterplot(abs(geneModuleMembership[moduleGenes, column]),

                   abs(geneTraitSignificance[moduleGenes, 1]),

                   xlab = paste("Module Membership in",
module,"module"),

                   ylab = "Gene significance for npi",

                   main = paste("Module membership vs. gene
significance\n"),

                   cex.main = 1.2, cex.lab = 1.2, cex.axis = 1.2,
col = module)
```

These below scripts help detect significant genes (high Gene significance and high Module membership) and the top 5 hub genes in each module (the blue module is an example, the others can be done similarly), respectively.

```
#identifying genes with high GS and MM

intra_modular_analysis=data.frame(abs(geneModuleMembership[moduleGen
es, column]),abs(geneTraitSignificance[moduleGenes, 1]))
rownames(intra_modular_analysis)=colnames(t(exp_dri_norm))[moduleCol
ors=="blue"] #only the blue module
View(intra_modular_analysis)

#high intramodular connectivity ~ high kwithin => hub genes (kwithin:
connectivity of the each driver gene in the turquoise module to all
other genes in the turquoise)
connectivity=intramodularConnectivity(adjacency, moduleColors)
connectivity=connectivity[colnames(t(exp_dri_norm))[moduleColors=="t
urquoise"],] #only the turquoise module
order.kWithin = order(connectivity$kWithin, decreasing = TRUE)
connectivity  =  connectivity[order.kWithin,]  #order  rows  following
kWithin
connectivity  =  connectivity[1:5,]  #top  5  genes  that  have  a  high
connectivity to other genes in the turquoise module
View(connectivity)
```

7. Patient stratification (Fig.1, Stage 2; Step 5)

7.1. Pre-processing clinical data & CNVs data

Source the R.script (1.CNA-preprocess.R) to process the two datasets: CNV data and clinical data.

```
#Load data objects and Data Preprocessing
source("1.CNA-preprocess.R")
```

7.2. Patient stratification by the identified driver genes

Source the R.script (2.CNA-hclut.R) to do this task that contains:

- As the same way described at the section 6.1, we find the best agglomeration method (i.e., Ward's method) using the function 'agnes' and realize that Ward's method outperforms the others again.
- The function 'hclust' generates a dendrogram tree of the patients by combining a dissimilarity matrix of all the patients, computed based on Euclidean distance by the function 'dist', with Ward's method, finding the relationship between all the patients.
- The function 'clValid' performs the Dunn's index (Fig.S11) and the average Silhouette width (Fig.S12) to determine how many patient groups are best. Collectively, Fig.S10-S12 imply that two groups are the best solution.
- The functions 'cutree' with 'agnes' distributes all the patients to each of two groups
- The function 'Heatmap' for the package ComplexHeatmap plots the result of patient stratification as a heatmap plot (Fig.S13).

```
#Patient stratification
source("2.CNA-hclut.R")
```

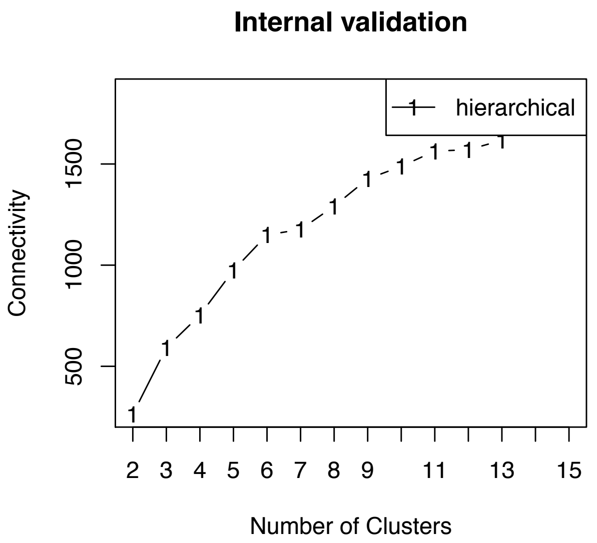

Figure S10 | Optimal group number detection. Two optimal groups were determined by the connectivity. The connectivity computes the degree of connectedness of a given group partitioning. The connectivity shows the connectedness of a given cluster partitioning and has a value between 0 and infinity. The user should choose a point reaching the most minimized value (y-axis).

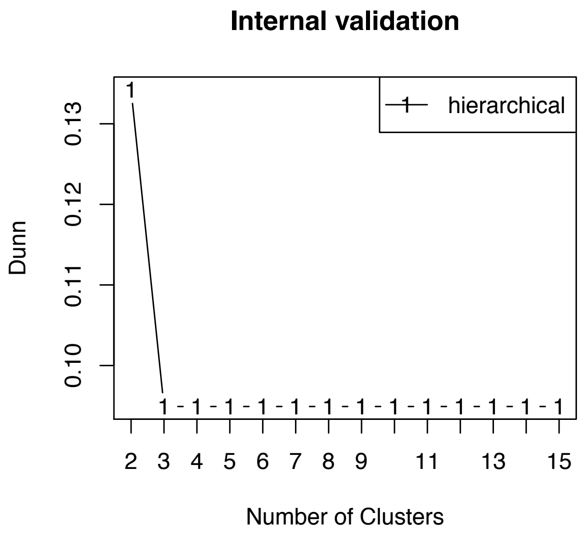

Figure S11 | Optimal group number detection. Two optimal groups were also determined by the Dunn's index. The Dunn's index (y-axis) has a value between zero (poorly clustered observations) and infinity (well clustered observations), and the place where the black line of Dunn's index plot peaks at, which implies that that group number is optimal.

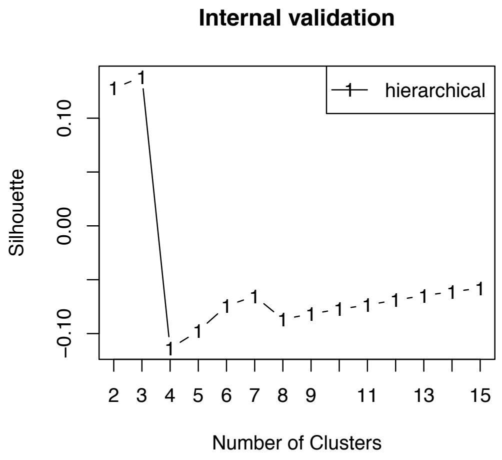

Figure S12. Optimal group number detection. Three optimal groups were determined by the Silhouette width. The average Silhouette has a value between -1 (poorly clustered observations) and 1 (well clustered observations), and the place where the black line of the Silhouette plot peaks at, which implies that that group number is optimal.

Collectively, Fig.S10-S12 show that the two number of groups is the best solution.

Then, we cut the discovered tree of patients above based on the optimal number of groups and visualize the result using a heatmap plot.

```
# agnes() with cutree() cuts the dendrogram into 2 groups
hc_a <- agnes(t(cna_dri), method = "ward")
```

```
sub_grp= cutree(as.hclust(hc_a), k = 2)

# Number of members in each cluster
table(sub_grp)
# sub_grp
# 1    2
# 993 1180
```

To show two groups on the top of the heatmap plot (Fig.S13), we must specify which patients belong to which identified groups.

```
> ## make a named vector from the vector
> info =as.data.frame(sub_grp)
> info$patient = rownames(info)
> info <-info[order(info$sub_grp),]
> info = dplyr::select(info,-patient)
> colnames(info) <- c('groups')
> info$groups = as.character(info$groups)
> cna_dri = cna_dri[,rownames(info)] #change the order of
> column/patients of cna data following the variable 'info'

> ## Heatmap annotation
> library(circlize)
> ha <- columnAnnotation(df = info, col = list(groups = c("1" =
"black", "2" = "green")))
```

And finally, we visualize the result as the heatmap plot (Fig.S13)

```
> #visualization using heatmap plot
> Heatmap(cna_freq, name = "CNA scale",
+         show_row_names = TRUE, show_column_names = FALSE,
+         row_dend_reorder = TRUE, column_dend_reorder = TRUE,
+         clustering_distance_rows = "euclidean",
+         clustering_distance_columns = "euclidean",
+         clustering_method_rows = "ward.D2",
+         clustering_method_columns = "ward.D2",
+         top_annotation = ha
+ )
```

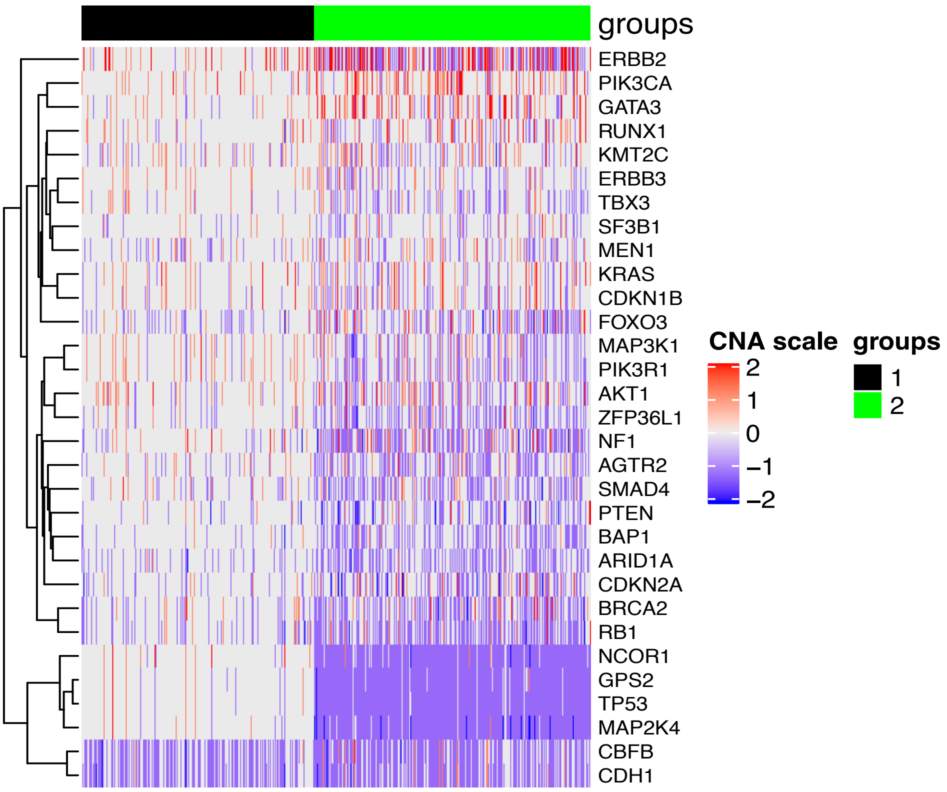

Figure S13. Differences in CNVs events between the identified groups. Clustering of BRCA patients based on the 31 driver genes. Two distinct groups are found (black and green). For CNA scale, Dark red, red, grey, blue and dark blue represent high-level amplification, amplification, copy-neutral, deletion and high-level deletion, respectively.

7.3. Comparision between the identified patient groups

We further perform the difference in the clinical features (i.e., survival rate, number of positive lymph nodes, Nottingham prognostic index, and cancer stage) between the two identified groups. Firstly, we must define the time and status of patients based on the clinical data.

```
#change the order of column/patients of clinical data following the
variable 'info'
cli = c_clinical; rownames(cli) = c_clinical$PATIENT_ID
grp=sub_grp; grp=as.data.frame(grp)
cli_surv =cli[rownames(grp),]
cli_surv = na_if(cli_surv,"") #transform "" (missing space) into NA
value
survData<-cbind(cli_surv$OS_MONTHS, cli_surv$OS_STATUS=="DECEASED")

#create survData
rownames(survData)<- rownames(cli_surv)
colnames(survData)<-c("time", "status")
survData <- as.data.frame(survData) #coxph and survfit require input
as data frame
```

Then, we now fit a Cox proportional hazards regression model and compute an estimate of a survival curve using ‘coxph’ and ‘survfit’, respectively. The function ‘ggsurvplot’ for the package survminer illustrates the Kaplan-Meier survival curves (Fig.S14).

```
coxFit1 <- coxph(
   Surv(time, status) ~ as.factor(sub_grp),
   data = survData,
   ties = "exact" )
pcox=round(summary(coxFit1)$logtest[3],8); pcox #p-value = 1.498e-05

mfit <- survfit(Surv(time, status == 1) ~ as.factor(sub_grp), data =
survData)

#visualization of survival rate between two groups
ggsurvplot(mfit, size=1,
            linetype = "strata",
risk.table = FALSE, fun ="pct", risk.table.col = "strata", break.x.by
= 50,
            xlab = "Time in months",
            legend = "bottom",
            legend.title = "Group", legend.labs = c("Group 1","Group
2"),
conf.int = TRUE, pval = paste("P-value",pcox,sep=" = " ), xlim =
c(0,200), palette = c("black", "green"))
```

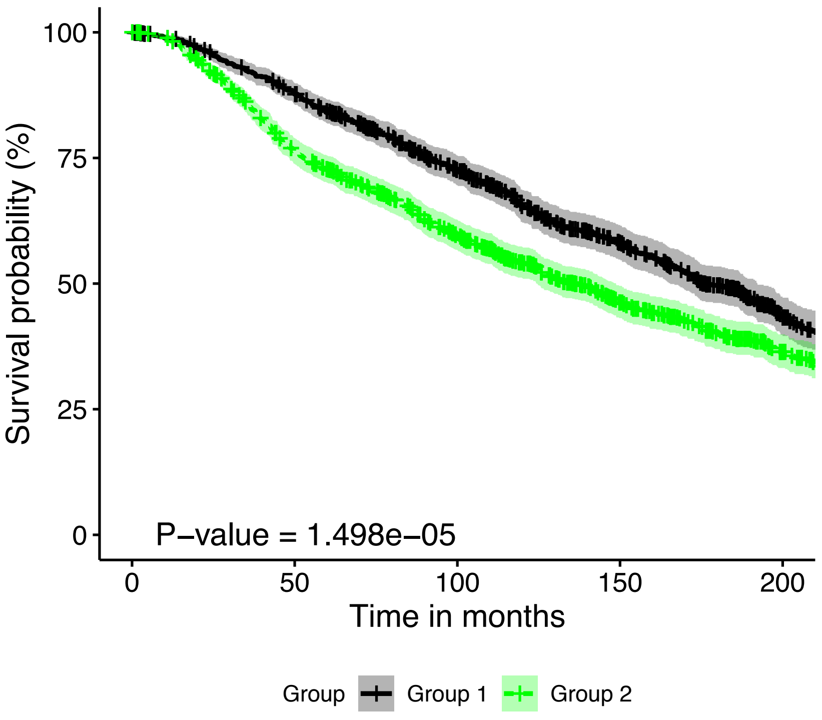

Figure S14. Difference in patient survival rates between the two groups.

Finally, we observe the differences between the two groups concerning the other clinical features.

```
library(dplyr)
library(ggplot2)
devtools::install_github("kassambara/ggpubr")
```

```
library("ggpubr")
library(ggsci)

#prepare data
a = info
cli_feature = c_clinical;
rownames(cli_feature) = cli_feature$PATIENT_ID;
cli_feature = cli_feature[rownames(info),]
a$lymph=cli_feature$LYMPH_NODES_EXAMINED_POSITIVE
a$npi=cli_feature$NPI
a$stage=cli_feature$stage

#comparison
install.packages("table1"); library(table1)
install.packages("compareGroups"); library("compareGroups")
#define specifically type of data
a$lymph = as.numeric(a$lymph); a$npi = as.numeric(a$npi)
a$stage = as.character(a$stage)
#start to perform comparisons
des=compareGroups::createTable(compareGroups::compareGroups(groups ~
., data = a, method = NA))
#save the results as xls file
compareGroups::export2xls(des, file = "tableSTAT.xlsx",
header.labels = c(p.overall = "p-value"))
```

For the number of positive lymph node (Fig.S15):

```
 set.seed(216)
#lymph nodes
p=ggboxplot(a, x = "groups", y = "lymph",
          color = "groups", palette = c("black", "green"),
          ylab = "Number of positive lymph nodes", xlab = "Groups",
          title = "Wilcoxon, P-value = 0.031") + border("black")
ggpar(p,legend="right",legend.title = "Groups")
```

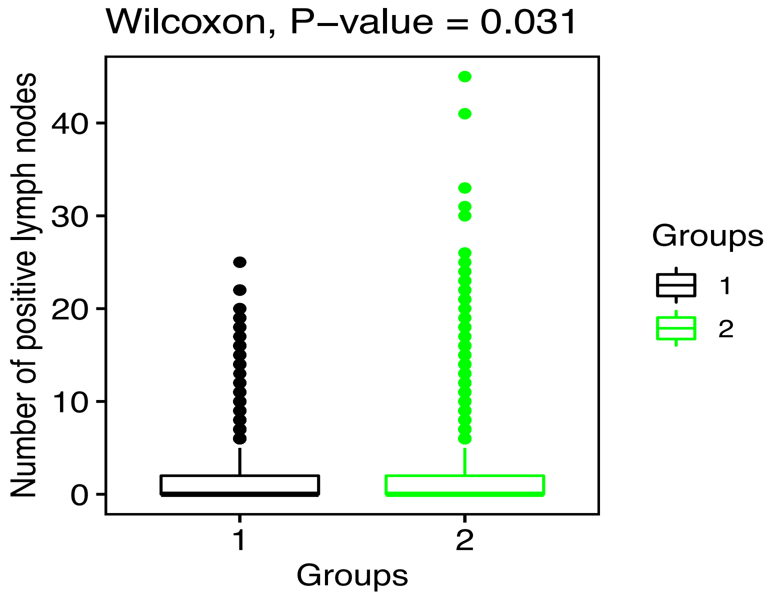

Figure S15. Difference in lymph between the two groups.

For the Nottingham prognostic index (Fig.S16)

```
#NPI
p1=ggboxplot(a, x = "groups", y = "npi",
            color = "groups", palette = c("black", "green"),
            ylab = "Nottingham prognostic index", xlab = "Groups",
            title = "Wilcoxon, P-value < 0.001") + border("black")
ggpar(p1,legend="right",legend.title = "Groups")
```

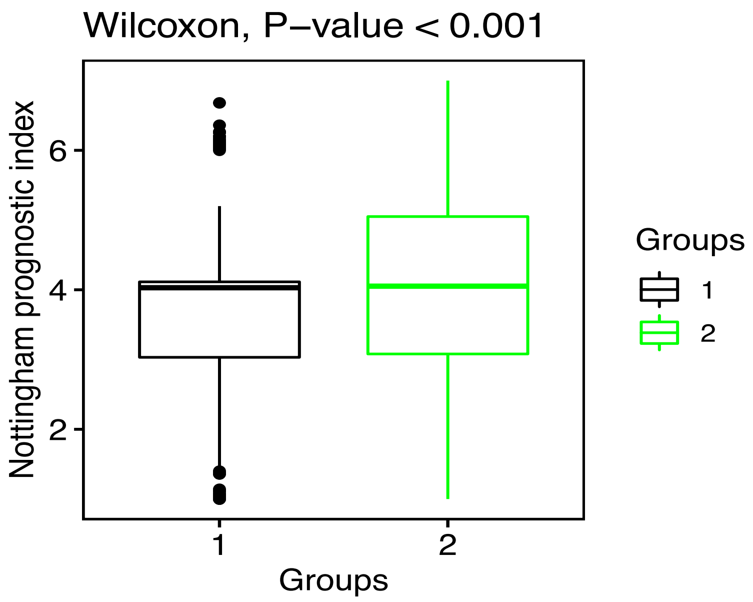

Figure S16. Difference in npi between the two groups.

And finally, for the tumor stage (Fig.S17)

```
 #stage
a_st = a %>% group_by(groups, stage) %>% summarise(patient.num = n())
## Create a column "patient.num" which is number of patients in each
combination of group and stage
a_st$Groups=as.character(a_st$groups)
a_st$stage=as.character(a_st$stage)
a_st=na.omit(a_st)
#stage
a_st = a %>% group_by(groups, stage) %>% summarise(patient.num =
n()) ## Create a column "patient.num" which is number of patients in
each combination of group and stage
a_st$Groups=as.character(a_st$groups)
a_st$stage=as.character(a_st$stage)
a_st=na.omit(a_st)
p2=ggplot(a_st, aes(x = Groups, y = patient.num, fill = stage)) +
#stacked barplot
  geom_bar(position = "fill", stat = "identity") +
theme(panel.background = element_blank()) +
  ggtitle("Chisq, P-value = 0.016") +
  scale_y_continuous(labels = scales::percent_format())
```

```
p2 + xlab("Groups") + ylab("Percent of patients")
```

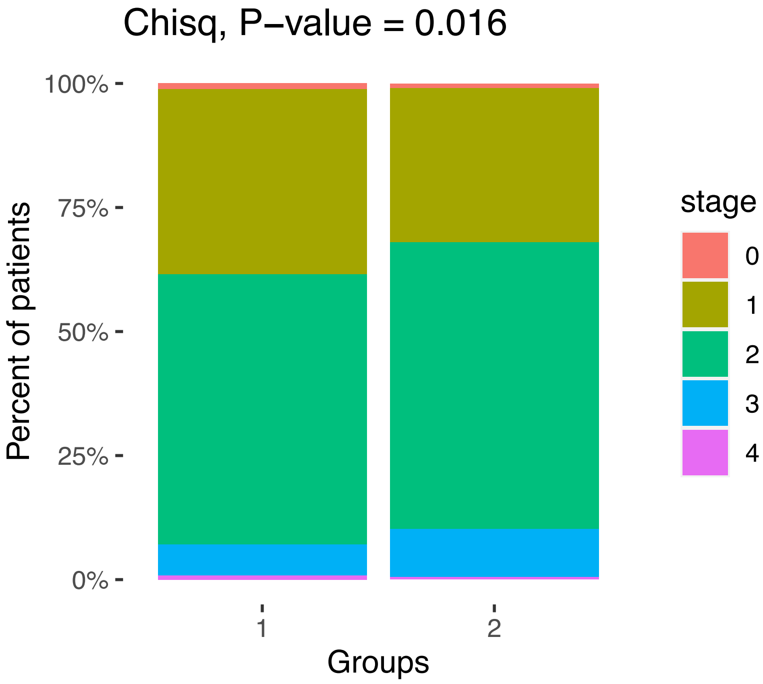

Figure S17. Difference in tumor stage between the two groups. Abbreviation: Chisq, Pearson's χ^2 test

8. References

1 Cancer Genome Atlas Research, N. *et al.* The Cancer Genome Atlas Pan-Cancer analysis project. *Nature genetics* **45**, 1113-1120, doi:10.1038/ng.2764 (2013).

2 Cerami, E. *et al.* The cBio Cancer Genomics Portal: An Open Platform for Exploring Multidimensional Cancer Genomics Data. *Cancer Discovery* **2**, 401-404, doi:10.1158/2159-8290.cd-12-0095 (2012).

3 Pereira, B. *et al.* The somatic mutation profiles of 2,433 breast cancers refine their genomic and transcriptomic landscapes. *Nature Communications* **7**, 11479, doi:10.1038/ncomms11479 (2016).

4 Arnedo-Pac, C., Mularoni, L., Muiños, F., Gonzalez-Perez, A. & Lopez-Bigas, N. OncodriveCLUSTL: a sequence-based clustering method to identify cancer drivers. *Bioinformatics* **35**, 4788-4790, doi:10.1093/bioinformatics/btz501 (2019).

5 Mularoni, L., Sabarinathan, R., Deu-Pons, J., Gonzalez-Perez, A. & López-Bigas, N. OncodriveFML: a general framework to identify coding and non-coding regions with cancer driver mutations. *Genome Biology* **17**, 128, doi:10.1186/s13059-016-0994-0 (2016).

6 Kircher, M. *et al.* A general framework for estimating the relative pathogenicity of human genetic variants. *Nature Genetics* **46**, 310-315, doi:10.1038/ng.2892 (2014).

7 Li, J. *et al.* Application of Weighted Gene Co-expression Network Analysis for Data from Paired Design. *Scientific Reports* **8**, 622, doi:10.1038/s41598-017-18705-z (2018).
